# Limited echocardiogram acquisition by novice clinicians aided with deep learning: A randomized controlled trial

**DOI:** 10.1093/biomethods/bpaf083

**Published:** 2025-11-07

**Authors:** Andre Kumar, Evan Baum, Caitlin Parmer-Chow, John Kugler

**Affiliations:** Department of Medicine, Stanford University School of Medicine, 300 Pasteur Drive, Stanford, CA 95401, United States; Department of Medicine, Stanford University School of Medicine, 300 Pasteur Drive, Stanford, CA 95401, United States; Department of Medicine, Stanford University School of Medicine, 300 Pasteur Drive, Stanford, CA 95401, United States; Department of Medicine, Stanford University School of Medicine, 300 Pasteur Drive, Stanford, CA 95401, United States

**Keywords:** bedside ultrasound, point-of-care ultrasound, competency, technique, patient care, medical education, echocardiogram, deep learning, artificial intelligence, neural network

## Abstract

The global shortage of sonographers has created significant barriers to timely ultrasound diagnostics across medical specialties. Deep learning (DL) algorithms have potential to enhance image acquisition by clinicians without formal sonography training, potentially expanding access to crucial diagnostic imaging in resource-limited settings. This study evaluates whether DL-enabled devices improve acquisition of multi-view limited echocardiograms by healthcare providers without previous cardiac ultrasound training. In this single-center randomized controlled trial (2023–2024), internal medicine residents (*N* = 38) without prior sonography training received a portable ultrasound device with (*N* = 19) or without (*N* = 19) DL capability for a two-week clinical integration period during regular patient care on hospital wards. The DL software provided real-time guidance for probe positioning and image quality assessment across five standard echocardiographic views. The primary outcome was total acquisition time for a comprehensive five-view limited echocardiogram (parasternal long axis, parasternal short axis, apical 4-chamber, subcostal, and inferior vena cava views). Assessments occurred at randomization and after two weeks using a standardized patient. Secondary outcomes included image quality using a validated assessment tool and participant attitudes toward the technology. Baseline scan times and image quality scores were comparable between groups. At two-week follow-up, participants using DL-equipped devices demonstrated significantly faster total scan times (152 s [IQR 115–195] versus 266 s [IQR 206–324]; *P* < 0.001; Cohen’s *D* = 1.7) and superior image quality with higher modified RACE scores (15 [IQR 10–18] versus 11 [IQR 7–13.5]; *P* = 0.034; Cohen’s *D* = 0.84). Performance improvements were most pronounced in technically challenging views. Both groups reported similar levels of trust in DL-functionality. Ultrasound devices incorporating deep learning algorithms significantly improve both acquisition speed and image quality of comprehensive echocardiographic examinations by novice users. These findings suggest DL-enhanced ultrasound may help address critical gaps in diagnostic imaging capacity by enabling non-specialists to acquire clinically useful cardiac images.

## Introduction

Access to diagnostic ultrasound imaging faces significant challenges due to critical shortages in the sonographer workforce, resulting in delayed examinations across multiple clinical disciplines worldwide [1–3]. In the United States alone, demand for ultrasound services has increased by 55.1% over the past decade, while educational capacity has expanded by only 23.0% [3]. This growing disparity necessitates innovative approaches to enhance patient access to diagnostic imaging. Potential solutions include technology-assisted acquisition methods, training of auxiliary healthcare personnel, and artificial intelligence (AI) enhanced systems to support non-specialist users [1, 4, 5]. Clinicians can also address this gap through the expanded use of point-of-care ultrasound (POCUS) to make expedient decisions on patient triage, risk stratification, or the need for more extensive diagnostic testing [6–8]. However, the acquisition of diagnostic-quality images, particularly in cardiac applications, remains challenging for providers without specialized training.

Recent advances in deep learning (DL), a sophisticated branch of machine learning where computer systems learn to identify patterns through multiple processing layers, have demonstrated remarkable capabilities in both facilitating image acquisition and enhancing interpretation of ultrasound studies [1, 5, 9, 10]. These algorithms can be deployed on ultra-portable devices connected to smartphones or tablets, enabling diagnostic assessments in settings with traditional access barriers [6, 7]. DL may assist healthcare workers in acquiring ultrasound images suitable for remote interpretation by specialists or provide real-time diagnostic support at the bedside [11].

Previous investigations have shown that DL-assisted ultrasounds can help inexperienced users obtain diagnostic-quality cardiac images, accurately assess ventricular function, and identify pericardial effusions [12, 13]. Additional applications include DL-augmented POCUS for diagnosing pediatric pneumonia [14], deep venous thrombosis [15], and pneumothorax [16]. While evidence suggests that non-sonographers can acquire diagnostic-quality images with DL assistance [12, 13], randomized studies comparing educational outcomes and the development of competency remain limited [5]. Moreover, existing studies often focus on single-view acquisitions with limited clinical utility [5], whereas clinically relevant examinations typically require multiple views for adequate diagnostic assessment.

This study addresses these knowledge gaps by evaluating whether DL-augmented portable ultrasound devices enhance novice scanning proficiency with limited echocardiograms that incorporate multiple standard views. We hypothesized that POCUS novices randomized to DL-enabled devices (Ultrasight^TM^) would demonstrate greater efficiency and technical proficiency across all standard cardiac views compared to those using conventional POCUS technology.

## Materials and Methods 

### Study population and setting

This randomized controlled trial was conducted at a single academic medical center from September 2023 through September 2024. Eligible participants included internal medicine residents rotating on inpatient general medicine services. We excluded residents who had previously completed the ultrasound elective offered within our residency program. The Stanford University Institutional Review Board approved this investigation, and the study was registered with ClinicalTrials.gov (Identifier: NCT05900440).

### Study design

**Figure 1:**
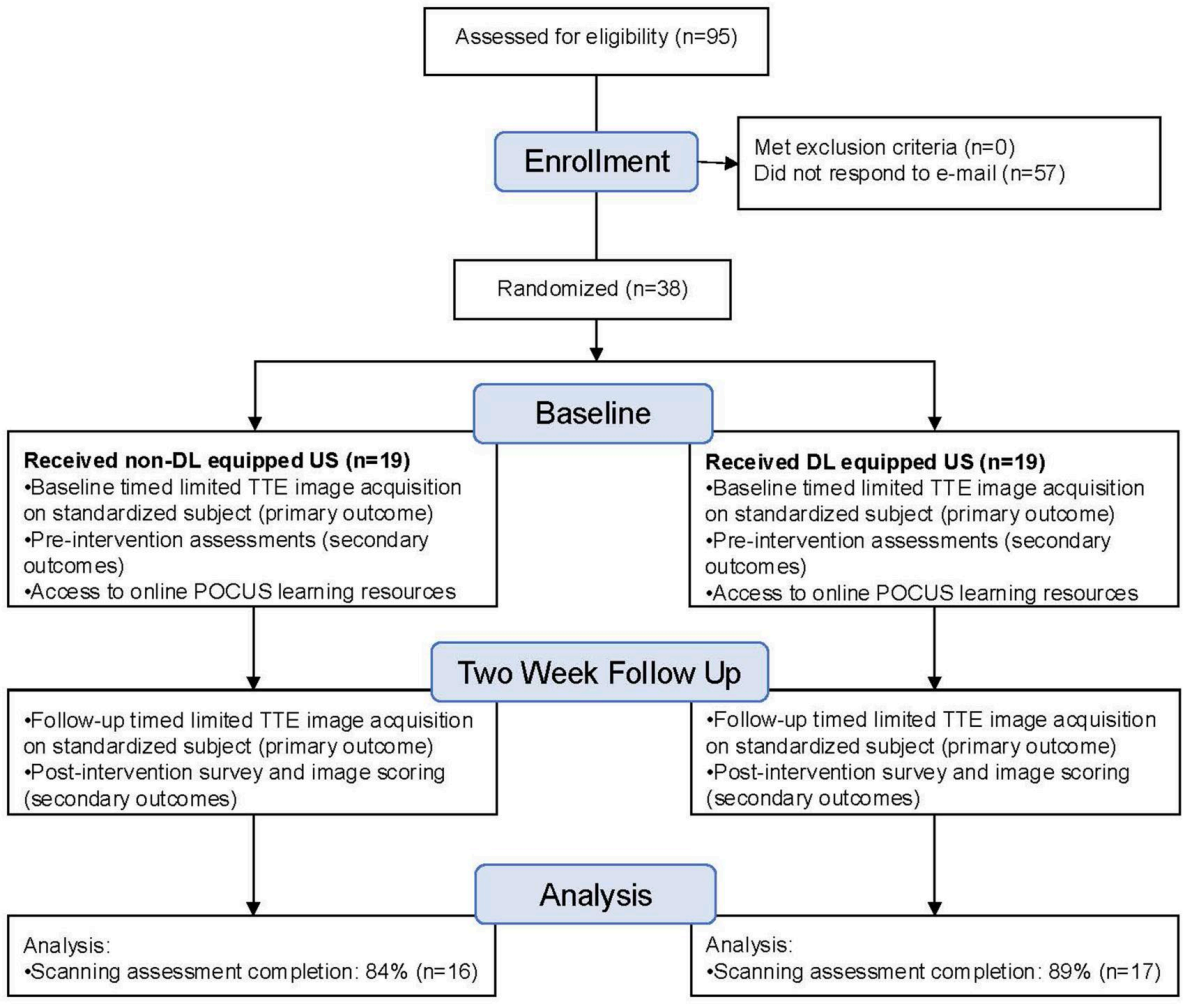
Study design. TTE, transthoracic echocardiogram; DL, deep learning

Participants (*N* = 38) were randomized in a 1:1 ratio to receive either a POCUS device with integrated DL-functionality (Lumify™ + Ultrasight™, *N* = 19) or a standard device without DL capabilities (Lumify™ alone, *N* = 19) for a two-week intervention period (Fig. 1). Outcomes were assessed at baseline randomization and at the conclusion of the two-week period.

Participants were permitted to use the assigned devices at their discretion during routine patient care activities while on service. All participants received standardized instruction on device functionality and were provided access to identical online educational resources regarding basic POCUS image acquisition and interpretation techniques (see Supplementary Material for detailed instructions that were provided to participants). To track utilization patterns, participants were instructed to save a representative image each time they used the device for any purpose. For patient confidentiality, these clinical images were not reviewed by the research team, and no feedback was provided on participants’ scanning technique during the intervention period.

### Devices and deep learning software

**Figure 2:**
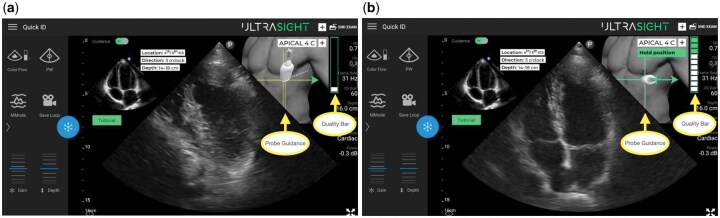
Deep learning (DL) software overlay. The system uses AI guidance that overlays over the standard Philips Lumify display to aid the user in image acquisition (yellow arrows). The figure shows two possible states of the system: navigation for an optimal view (**a**), and holding the position once an optimal view has been obtained (**b**). The display is continuously updated while the user performs the exam

This study utilized the Philips Lumify™ portable ultrasound platform for both study arms. Participants in the DL intervention group used the device with integrated Ultrasight™ software, which provides real-time guidance for optimal transducer placement to acquire five standard cardiac views: parasternal long axis (PLAX), parasternal short axis (PSAX), apical 4-chamber (A4C), subcostal (SC), and inferior vena cava (IVC). The system overlays directional guidance on the ultrasound display and provides real-time feedback on image quality (Fig. 2). The control group utilized identical Lumify™ hardware without the DL guidance features.

### Deep learning system architecture

The Ultrasight™ system employs a deep convolutional neural network utilizing a ResNet-50 backbone architecture for feature extraction from ultrasound video streams [17].

Detailed algorithm description, training methodology, and validation procedures are provided in the manufacturer’s validation publication and corresponding technical documentation in the Supplementary Material [17]. As a commercial, FDA-cleared system, full source code and training hyperparameters are proprietary and cannot be publicly disclosed; however, all accessible implementation details and validation metrics have been cited herein for transparency.

The network processes real-time B-mode ultrasound images at 15–30 frames per second and generates three primary outputs through specialized prediction heads: (1) view classification across the five cardiac views, (2) 6-degree-of-freedom probe positioning guidance providing directional cues for rotation and translation corrections, and (3) real-time image quality assessment based on anatomical landmark detection and optimal imaging plane alignment (Fig. 2).

### Training data and methodology

The Ultrasight™ algorithm was developed and validated on a multi-institutional dataset of >50 000 transthoracic echocardiogram studies (Supplementary Material) [17]. The dataset encompassed diverse patient demographics, pathologies, and imaging conditions, providing the foundation for generalizable performance across operator experience levels [17]. Data annotation was performed by board-certified cardiologists and experienced cardiac sonographers. The training employed transfer learning, initializing from ImageNet-pretrained weights and fine-tuning on cardiac ultrasound data using a multi-task learning approach with weighted loss functions for view classification (cross-entropy), positioning guidance (mean squared error), and quality assessment (ordinal regression). Data augmentation techniques included rotation, translation, brightness and contrast adjustment, and simulated probe motion artifacts to improve model robustness.

### Validation performance

On held-out validation datasets, the view-classification module achieved >90% accuracy across all five standard cardiac views and the image-quality prediction correlated strongly with expert ratings (*r* = 0.82), consistent with the performance reported in the independent multicenter validation study (Supplementary Material) [17].

### System operation

The system overlays directional guidance on the ultrasound display and provides continuous real-time feedback on image quality (Fig. 2). The DL system operates on standard B-mode ultrasound images with typical clinical settings (depth: 12–20 cm, frequency: 2–5 MHz). Importantly, the system does not require pre-specified image quality thresholds to function; rather, it is designed to guide users from suboptimal to optimal image quality across a spectrum of acquisition skill levels. Additional technical information can be found in the manufacturer’s documentation referenced in the Supplementary Material.

### Outcome measures

The primary outcome was the total time required to acquire a complete limited echocardiogram comprising all five standard views (PLAX, PSAX, A4C, SC, and IVC) at the two-week follow-up assessment. Doppler imaging was not included in the acquisition protocol. Secondary outcomes included the quality of acquired images at follow-up and participant attitudes toward the technology.

### Assessment procedures

Standardized assessments were conducted at randomization (baseline) and after the two-week intervention period. To evaluate skill transferability rather than device-specific familiarity, all assessments were performed using a different ultrasound system (Butterfly iQ+) than those provided during the intervention period. All scanning evaluations were performed on the same standardized patient to ensure consistency of acoustic windows given our outcomes included time spent scanning and acquired image quality.

A study investigator was present during assessments to provide standardized instructions and manage the assessment protocol (Supplementary Material), but they did not observe or comment on the images being acquired. For timing measurements, participants verbally indicated when they had acquired each view, and time was measured from the moment of initial probe contact until they notified the proctor they had acquired the view. Participants then saved a 5-s video clip of each view for subsequent quality evaluation. If participants were unable to obtain a particular view after reasonable effort, they could indicate this to the proctor, and these instances were recorded as incomplete acquisitions. Participants could spend unlimited time attempting to acquire a particular view.

Image quality was assessed using the modified Rapid Assessment of Competency in Echocardiography (RACE) scale, which rates each view on a 1–5 ordinal scale (details provided in Supplementary Materials). This validated assessment tool has demonstrated excellent interrater reliability (α = 0.87) in previous studies evaluating POCUS image acquisition quality [17–19]. Three reviewers (AK, EB and JK), who were blinded to the study arms, independently reviewed the baseline and follow-up assessment scans to assign RACE scores. Composite RACE scores were calculated by averaging ratings across all three reviewers.

Participant surveys were administered at the conclusion of the study period to assess attitudes toward POCUS, trust in the DL system, and self-reported confidence in image acquisition. Attitudes were measured using standardized 5-point Likert scales based on previously validated assessment instruments [20, 21].

### Statistical analysis

A priori power analysis determined that 38 participants (19 per group) would provide >80% power to detect a 30% difference in the primary outcome between groups (Cohen’s *D* = 0.73, two-sided alpha = 0.05). These assumptions were based on unpublished data from previous investigations at our center, as well as recent studies of AI-assisted cardiac imaging and our experience teaching cardiac POCUS to novice operators [5, 20, 21]. Within the limitations of these assumptions, this study was sufficiently powered to detect a difference in the primary outcome.

Baseline characteristics and performance metrics were compared between groups using appropriate statistical tests. Scan time distributions were visualized with box/violin plots. We report median and interquartile range (IQR) by randomized group and employed Mann-Whitney U testing to compare medians at follow-up. Similar analyses were conducted for all secondary outcomes. Cohen’s *D* was calculated to measure effect size for significant differences, and Chi-square tests were used to compare categorical variables such as confidence levels.

All statistical analyses were performed using Python (Python Software Foundation, 2024), with specialized packages including NumPy, pandas, and SciPy [22–24].

## Results

### Participant characteristics

From a pool of *N* = 95 eligible residents, *N* = 38 volunteered and were enrolled in the study. No residents required exclusion. Randomization resulted in *N* = 19 participants assigned to the DL-enabled device group and *N* = 19 to the standard device group. Completion rates at two-week follow-up were high, with *N* = 33 residents (87%) completing the final assessment, including *N* = 17 (89%) in the DL group and *N* = 16 (84%) in the non-DL group (Table 1). Demographic characteristics and baseline POCUS experience were similar between groups (Table 1). Both groups documented comparable frequency of device usage during the intervention period as recorded in electronic logs (DL mean 5.4 times [SD 3.2] versus non-DL mean 4.8 times [SD 2.9]; *P* = 0.59).

**Table 1.** Participant demographics.^a^

	Baseline DL	Baseline non-DL	Post-intervention DL	Post-intervention non-DL
**Completed scanning assessments (%)**	19 (100%)	19 (100%)	17 (89%)	16 (84%)
**PGY-1 (%)**	6 (32%)	6 (32%)	6 (35%)	6 (38%)
**PGY-2 (%)**	8 (42%)	7 (37%)	7 (41%)	4 (25%)
**PGY-3 (%)**	5 (26%)	6 (32%)	4 (24%)	6 (38%)
**Females (%)**	9 (47%)	9 (47%)	8 (47%)	7 (44%)
**Exposure to prior POCUS courses (%)**	4 (21%)	2 (11%)	3 (18%)	3 (19%)

a PGY, post-graduate year; POCUS, point-of-care ultrasound.

### Primary outcome: limited echocardiogram acquisition time

At baseline, both groups demonstrated statistically similar times to acquire a complete five-view limited echocardiogram (DL median 239 s [IQR 146–267] versus non-DL median 264 s [IQR 151–332]; *P* = 0.31). At the two-week follow-up assessment, the DL group demonstrated significantly faster scanning times with a large effect size (median 151 s [IQR: 115–195]) compared to the non-DL group (median 267 s [IQR: 206–325]; *P* < 0.001; Cohen’s *D* = 1.7; Table 2).

**Table 2.** Study outcomes.^a^

	Overall	DL group	Non-DL group	*P*-value
**Time to acquire view, median seconds (IQR)**				
**Overall**	204 (152–291)	151 (115–195)	267 (206–325)	**<.001**
**PLAX**	44 (20–57)	23 (14–34)	56 (49–68)	**<.001**
**PSAX**	26 (16–50)	20 (14–39)	37 (17–54)	.36
**A4C**	53 (38–86)	39 (32–48)	84 (58–97)	**<.001**
**SC**	33 (19–49)	31 (20–46)	37 (19–65)	.28
**IVC**	27 (19–41)	26 (18–35)	27 (19–43)	.77
**Modified RACE score, median (IQR)**				
**Overall**	12 (8–16)	15 (10–18)	11 (7–14)	**.034**
**PLAX**	3 (1–3)	3 (2–3)	2 (1–3)	.14
**PSAX**	2 (2–3)	3 (2–4)	2 (1–2)	**.015**
**A4C**	2 (1–3)	3 (1–3)	1 (1–2)	**.038**
**SC**	3 (1–3)	3 (1–3)	3 (1–3)	.55
**IVC**	3 (1–4)	3 (1–4)	3 (2–4)	.12
**Device usage rates, mean (SD)**	5.1 (3.2)	5.4 (3.2)	4.8 (2.9)	.59

a PLAX, parasternal long axis; PSAX, parasternal short axis; A4C, apical four chamber; SC, subcostal axis; IVC, inferior vena cava axis; DL, deep learning; IQR, interquartile range. Bolded values denote statistical significance.

This performance difference was primarily driven by faster acquisition of two technically challenging views: the PLAX view (DL median 23 s [IQR 14–34] versus non-DL median 56 s [IQR 49–68]; *P* < 0.001, Cohen’s *D* = 1.5) and the A4C view (DL median 39 s [IQR 32–48] versus non-DL median 84 s [IQR 57–97]; *P* < 0.001; Cohen’s *D* = 1.1). The remaining views (PSAX, SC, and IVC) showed no significant differences in acquisition times between groups at follow-up (Table 2).

### Secondary outcomes

#### Image quality

At baseline, both groups demonstrated statistically similar median RACE scores (DL median 13 points [IQR 8.5–14.5] versus non-DL group median 11 points [IQR: 8.5–15]; *P* = 0.98). At follow-up, the DL group achieved significantly higher median RACE scores (15 points [IQR 10–18]) versus the non-DL group (11 points [IQR 7–13.5]; *P* = 0.034; Cohen’s *D* = 0.84; Table 2).

The quality differences were most pronounced in the PSAX view (DL median 3 points [IQR 2–4] versus non-DL median 2 points [IQR 1–2]; *P* = 0.014; Cohen’s *D* = 0.99) and A4C view (DL median 3 points [IQR 1–3] versus non-DL median 1 point [IQR 0.5–2]; *P* = 0.038; Cohen’s *D* = 0.87). Other views did not show statistically significant differences in RACE scores between groups (Table 2).

#### Scan completion rates

At baseline, both groups had statistically similar rates of incomplete views (DL: 18 instances versus non-DL: 4 instances; Chi-Square 1.42, *P* = 0.23). At follow-up, the DL group had significantly lower failure rates (DL: 1 instance versus non-DL: 8 instances; Chi-Square 5.77; *P* = 0.016). Failure rates by specific view at follow-up were: PLAX (3.2%), PSAX (3.2%), A4C (16%), SC (3.2%), and IVC (3.2%).

#### Participant attitudes

Among the *N* = 20 participants (53%) who completed the optional post-intervention survey, half (50%) agreed or strongly agreed they would generally trust DL functionality on ultrasound devices. Additionally, 70% expressed trust in real-time guidance for probe positioning using DL, and 70% would trust auto-interpretation features of DL on ultrasound machines.

## Discussion

As artificial intelligence applications in medical imaging continue to evolve, DL-enabled ultrasound systems offer potential to expand access to diagnostic testing, particularly in underserved regions, by enabling non-specialists to acquire clinically useful imaging studies [1, 5, 10, 12, 13, 25, 26]. This randomized trial demonstrated that novice ultrasound users equipped with DL-enhanced devices could obtain comprehensive limited echocardiograms more efficiently and with higher image quality suitable for diagnostic interpretation compared to users of standard devices.

Our findings extend and complement existing research on DL-assisted ultrasound imaging in several important ways.

Previous investigations have demonstrated that DL-assisted ultrasound can help inexperienced users obtain diagnostic-quality cardiac images and accurately assess ventricular function and pericardial effusions, but randomized trials have been scarce [5, 12, 14]. Earlier work demonstrated DL's utility for single cardiac view acquisition [5], but our study confirms its effectiveness for more comprehensive, clinically relevant examinations such as the limited echocardiogram which require multiple views. Notably, Narang et al. (2021) showed that novices using Caption Health’s AI guidance system (a different commercial platform) could acquire single-view echocardiograms comparable in quality to those obtained by experienced sonographers [12, 14]. Our study builds upon this work by: (1) employing different hardware and software platforms (Philips Lumify with Ultrasight), suggesting these benefits may be generalizable across technological implementations; (2) extending beyond single-view acquisition to comprehensive five-view limited echocardiograms with greater clinical utility; and (3) implementing a randomized controlled design with standardized outcome assessments.

Our findings also contribute to understanding different approaches to DL-enhanced ultrasound. While some systems focus on fully automated scanning with minimal operator input, our study demonstrates that real-time guidance enabling active operator engagement can effectively improve skill acquisition. This guided approach may offer advantages for clinical education by helping users develop transferable scanning skills rather than dependence on specific automated systems. Previous research with conventional ultrasound devices has established that approximately 20–30 scanning sessions are typically required to achieve basic competency in cardiac POCUS [17, 20, 27–29]. Notably, participants in our study performed substantially fewer scans (approximately 5 per group) during the intervention period, yet the DL-group still demonstrated significantly superior performance in both acquisition time and image quality. In contrast, our prior work has shown that simply providing novices with ultrasound devices without intelligent guidance does not meaningfully improve scanning proficiency [21]. These results suggest that DL-enabled technologies may accelerate the learning curve for ultrasound skills, potentially offering alternatives to traditional teaching methods that are difficult to scale due to faculty constraints [30–32].

The performance advantages in our study were primarily observed in the technically challenging PLAX and A4C views, which often prove most difficult for novice scanners due to their dependence on patient body habitus, precise positioning, and optimal scanning angles [21]. Similarly, image quality improvements were most prominent in the PSAX and A4C views, which are particularly susceptible to off-axis acquisition that can rapidly degrade diagnostic value. The magnitude of these differences, as reflected in the large effect sizes, suggests clinically meaningful improvements that could enhance diagnostic capability when implemented in clinical settings. There can also be an argument on whether the statistically significant differences in scan times and their effect sizes would result in clinically meaningful differences for patients or providers. We would suggest that any improvement in scan time that does not compromise image quality supports the feasibility of this technology to be widely implemented across non-traditional environments to improve patient access.

Our assessment protocol allowed participants unlimited time to attempt each view, with self-reported inability constituting a failed acquisition. While this approach respects individual variation in persistence and reduces arbitrary time constraints that might penalize slower but ultimately successful learners, it introduces variability based on individual determination rather than purely technical ability. A standardized time threshold (e.g. 120 s maximum per view) might provide more objective failure criteria, though it risks penalizing thorough, methodical learners. The very low failure rates observed at follow-up in the DL group (1.2%) compared to the control group (9.6%) suggest that the DL system enabled most users to acquire views within reasonable timeframes, regardless of individual persistence patterns. Nevertheless, future studies should consider predetermined time limits to standardize failure criteria.

This study has several important limitations that warrant consideration. First, it was conducted at a single academic institution with internal medicine residents, which limits generalizability to other clinical settings, healthcare systems, and provider types. The DL system used in this study was trained on a large, multi-site dataset (greater than 50 000 studies) with demographic and clinical diversity intended to support broad generalization. However, our assessments were performed on a single standardized patient with adequate acoustic windows. These findings may not fully reflect performance in patients with challenging body habitus, significant cardiac pathology, or other anatomical variations that complicate image acquisition. Future multicenter studies across varied patient populations and clinical contexts are needed to establish broader generalizability.

Second, while our initial sample size calculation used parametric assumptions, the primary analysis employed non-parametric tests due to non-normal data distribution, which may have affected statistical power. Although we achieved our target enrollment and observed large effect sizes, the modest sample size limits our ability to detect smaller effects and increases susceptibility to baseline imbalances.

Third, participants were not blinded to their study arm assignment, which could introduce performance bias. Users in the DL group may have demonstrated increased confidence or persistence knowing they had AI-assistance. However, the objective nature of our primary outcomes (timed acquisition and blinded image quality assessment) partially mitigates this concern, as participants could not directly influence the RACE scores assigned by blinded assessors. The magnitude of observed effect sizes (Cohen’s *D* = 1.7 for acquisition time) suggests that performance differences extended beyond purely motivational factors.

Fourth, although baseline incomplete view rates did not reach statistical significance (*P* = 0.23), the absolute difference between groups (DL: 18 failed views versus non-DL: four failed views) was notable and may reflect chance imbalance. The marked improvement in completion rates demonstrated by the DL group at follow-up (from 18 baseline failures to 1 follow-up failure) compared to the control group (from 4 to 8 failures) suggests the intervention effect remained robust despite this baseline difference.

Fifth, our study focused on clinical and educational outcomes rather than comprehensive AI-specific performance metrics such as guidance accuracy, false positive rates for view detection, or detailed interaction analytics. Future studies should incorporate such metrics to better characterize AI system behavior and identify areas for algorithmic improvement.

Sixth, while this study provides quantitative outcomes using a commercial DL platform, we acknowledge the partial limitation in reproducibility due to restricted access to proprietary training code and hyperparameters. We have mitigated this by referencing all publicly available manufacturer documentation and independent validation studies to allow external verification of algorithm design and performance characteristics.

Finally, the short two-week follow-up period limits conclusions about long-term competency development and skill retention. Longer-term studies examining whether DL-assisted learning leads to sustained competency or whether users become dependent on AI guidance would be valuable.

## Conclusion

Healthcare providers without specialized sonography training who were randomized to use DL-enabled ultrasound devices for a brief intervention period demonstrated significantly faster image acquisition and higher image quality scores for comprehensive limited echocardiograms compared to those using conventional devices. These findings suggest that DL-enhanced ultrasound technology may accelerate skill acquisition for novice users, though the magnitude of benefit in diverse clinical settings and patient populations requires further investigation. As DL capabilities continue to advance, future multicenter studies should evaluate the level of proficiency achievable by novice operators across varied contexts, the durability of learned skills, and whether DL-assisted imaging by non-specialists can effectively expand diagnostic access in resource-limited settings while maintaining diagnostic accuracy and clinical utility.

## Supplementary Material

bpaf083_Supplementary_Data

## Data Availability

The statistical analysis plan, code, and datasets generated and/or analyzed during the current study are not publicly available due to sharing limitations from our IRB protocol, but are available from the corresponding author on reasonable request.

## Supplementary data

*Conflict of interest statement.* Dr Kumar reports receiving consultant fees from Caption Health, which is unrelated to this body of work. The other authors do not have any financial interests to disclose. This study was an investigator-initiated study. None of the device manufacturers used in this study oversaw, influenced, or reviewed the data presented prior to submission. The authors vouch for the independent nature by which this investigation was conducted and disseminated, without influence from outside organizations.
