# Supplementary material for: Limited echocardiogram acquisition by novice clinicians aided with deep learning: A randomized controlled trial
Source: Biol Methods Protoc. 2025 Nov 7;10(1):bpaf083. doi: 10.1093/biomethods/bpaf083 (PMC12627401; doi:10.1093/biomethods/bpaf083)
Supplement: bpaf083_Supplementary_Data [file bpaf083_supplementary_data.zip › UltraSight-AI-Guidance-User-Manual-ver1.9-.pdf]

ULTRASIGHT

UltraSight AI Guidance

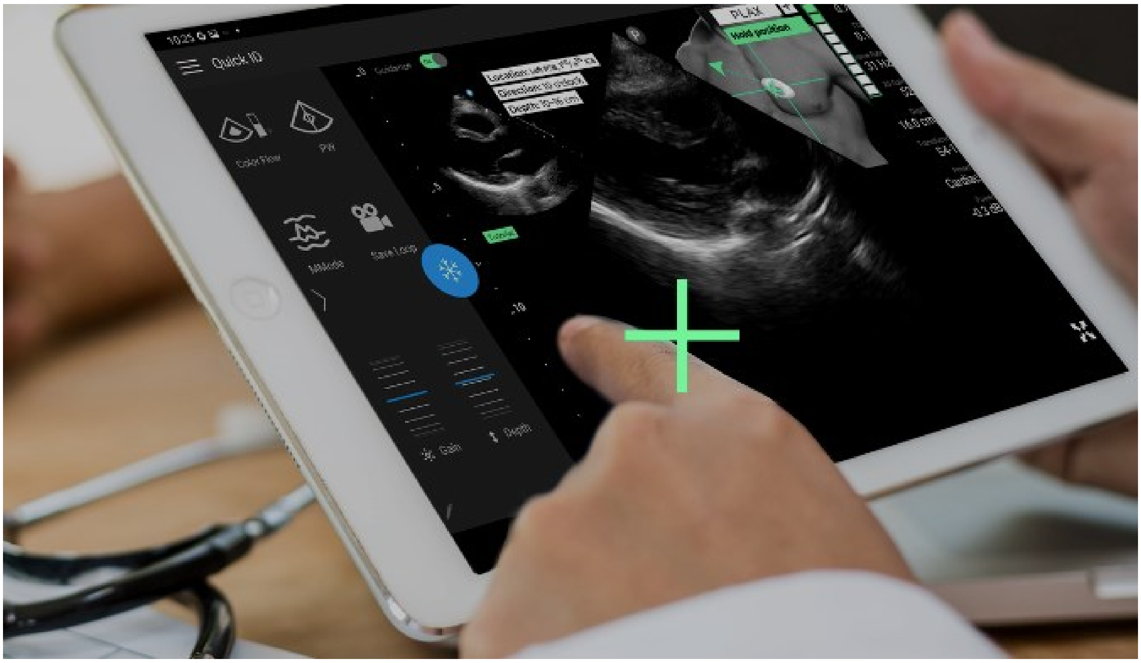

User Manual

Document No.: UM-0001
Revision 1.9, June, 2024

Copyright © 2022 UltraSight Ltd. All rights reserved.

This document is intended to be used by customers and licensed to them as part of their UltraSight product purchase or to meet regulatory commitments as required by the FDA under 21 CFR 892.2050 (and any amendments to it) and other local regulatory requirements. Use of this document by unauthorized persons is strictly prohibited.

No part of this publication may be reproduced, stored in a retrieval system, or transmitted in any form and by any means, electronic, mechanical, photo reproductive, recording, or otherwise, without the express prior written permission of UltraSight Ltd.

UltraSight Ltd. reserves the right to change or improve its products and accompanying technical literature without prior notice of changes or improvements.

The marks contained herein represent some of the trademarks or registered trademarks of UltraSight Ltd. or its subsidiaries and affiliates in the United States and/or other countries. Unless otherwise noted, all materials are protected as the copyrights, trade dress, trademarks, and/or other intellectual properties owned by UltraSight Ltd. and/or its subsidiaries and affiliates or by other parties that have licensed their material to UltraSight Ltd. All rights not expressly granted are reserved.

No part of this manual may be reproduced or copied in any form and by any means – graphic, electronic, mechanical, magnetic, optical, chemical, including photocopying, typing, translated into any language or computer language, or information retrieval systems – without the written permission of UltraSight Ltd.

All intellectual property rights, including those created by patent grant or registration of a utility model or a design, are expressly reserved. Any unauthorized use is illegal.

© 2022 UltraSight Ltd. All rights reserved. UltraSight™ and the UltraSight logo are trademarks of UltraSight Ltd. and are registered in the U.S. and other countries.

MD

UltraSight Ltd.
Pinhas Sapir 8 st, Scientific Park,
PO Box 594, Ness Ziona 7403631, Israel

Phone: +1-724-648-3514
Email: info@ultrasight.com
Web: www.ultrasight.com

EC REP Donawa Lifescience
Piazza Albania, 10
00153 Rome, Italy

Disclaimer

The UltraSight AI Guidance software should only be used in the manner specifically set forth in this user manual. Use of the UltraSight AI Guidance software in a manner not expressly described in this user manual (which may be updated from time to time by UltraSight) may lead to improper product performance, potential for hazard, and will void all warranties of any kind that are extended by UltraSight in connection therewith. The performance of the UltraSight AI Guidance software can only be assured under the following conditions: (i) The application has been used for its indicated use (see Indications for Use in this User Manual), (ii) this application has only been used in accordance with the operating instructions contained in this User Manual, (iii) all fittings, extensions, readjustments, changes required for the use of the UltraSight AI Guidance software have solely and exclusively been carried out by an UltraSight authorized representative, (iv) the user has been registered at UltraSight.

For medical questions, please consult a physician, and in the case of a medical emergency, immediately contact your local emergency services provider.

Neither UltraSight nor any of its principals, directors, employees, affiliates, representatives, and assigns shall be responsible in any way for any physical harm and/or property damage arising from the operation or use of either the UltraSight AI Guidance software or this user manual. Without limiting the foregoing, UltraSight makes no warranty of any kind regarding this user manual. In addition, UltraSight shall not be liable for errors contained in this user manual or for incidental or consequential damages in connection with the furnishing or use of any material contained within this user manual.

Accordingly, the content in this user manual does not and will not form part of any contract between UltraSight and any user of the UltraSight AI Guidance software. No person has the authority to bind UltraSight to any representation or warranty except as either specifically set forth above and/or in the UltraSight User Agreement.

The content of this user manual has been compiled for information purposes only and is subject to change by UltraSight without notice. Any modifications and amendments hereto will be deemed included in subsequent versions of the UltraSight user manual. In addition, the content in this user manual does not purport to cover all details or variations of the UltraSight AI Guidance software and does not address every possible contingency regarding the use of the UltraSight AI Guidance software.

This user manual contains proprietary information which is protected by international copyright. No part of this document may be photocopied, reproduced, or translated without the prior written consent of UltraSight. UltraSight is protected by the following patents: US11593638B2, EP3793447B1 and US11478226B2. Any unauthorized use of any information and content from the UltraSight user manual is illegal, and UltraSight Ltd. will prosecute violators.

Please read this user manual thoroughly and feel free to contact our product support team with any questions at: support@ultrasight.com.

License Agreement

All computer programs copyright 2022 by UltraSight Ltd. or its suppliers are licensed under the following software agreement.

UltraSight Ltd. or its suppliers retain(s) ownership of and title to any computer program supplied with the Device and the trade secrets embodied in such computer programs. Subject to the Buyer's acceptance and fulfillment of the obligations in this paragraph. UltraSight Ltd. grants the Buyer a personal, non-transferable, non-exclusive license to use any computer program supplied with the Device that is necessary to operate the Device solely on the medium in which such program is delivered for the purpose of operating the Device in accordance with the instructions set forth in the operator's manuals supplied with the Device and for no other purpose whatsoever. Buyer may not reverse-assemble, reverse-compile, or otherwise reverse-engineer such computer programs, nor may Buyer make a copy of such program or apply any techniques to derive the trade secrets embodied therein. In the event of a failure by Buyer to comply with the terms of this license, the license granted by this paragraph shall terminate. Further, because unauthorized use of such computer programs will leave UltraSight Ltd. without an adequate remedy at law, Buyer agrees that injunctive or other equitable relief will be appropriate to restrain such use, threatened or actual. Buyer further agrees that (i) any of UltraSight Ltd. suppliers of software is a direct and intended beneficiary of this end-user sublicense and may enforce it directly against Buyer with respect to software supplied by such supplier, and (ii) no supplier of ultrasight shall be liable to buyer for any general, special, direct, indirect, consequential, incidental or other damages arising out of the sublicense of the computer programs supplied with the device.

Table of Contents

CHAPTER 1 Introduction **1-1**

1.1 About this Manual 1-1

1.2 Conventions....1-1

1.3 Warnings 1-2

1.4 Cautions....1-4

1.5 Intended Purpose 1-5

1.6 Intended User 1-5

1.7 Indication for Use 1-5

1.8 Contraindications 1-6

1.9 Clinical Benefits and Precautions 1-7

1.9.1 Clinical Benefit....1-7

1.9.2 Precautions Regarding Specific Patient Types......1-7

1.10 Abbreviations and Acronyms....1-8

CHAPTER 2 Safety Considerations **2-1**

2.1 Introduction....2-1

2.2 Equipment Classification and Compliance 2-2

2.2.1 Classification 2-2

2.2.2 Electromechanical Safety Standards Met 2-2

2.2.3 Compliance 2-2

2.3 Biological Safety....2-2

CHAPTER 3 System Description **3-1**

3.1 Introduction....3-1

3.1.1 Medical Background 3-1

3.2 Device Description 3-3

3.2.1 General 3-3

3.2.2 Application Description....3-4

3.2.3 About the UltraSight AI Guidance Software 3-5

3.2.4 System Requirements 3-7
3.3 Device Labels 3-8

CHAPTER 4 The UltraSight Application 4-1
4.1 Installing the Application 4-1
4.2 Registration and Entitlement 4-2
4.3 Launching the UltraSight Application 4-2
4.4 The UltraSight Application 4-5
4.4.1 Main Menu 4-6
4.4.2 Views Menu 4-10
4.4.3 Guidance Switch 4-11
4.4.4 Static Guidance 4-12
4.4.5 Tutorial 4-13
4.4.6 Dynamic Real-Time Guidance 4-15
4.4.7 De-Brief Mode 4-16

CHAPTER 5 Ultrasound Scanning Operations 5-1
5.1 Acoustic Windows 5-1
5.2 Probe Initial Position 5-1
5.3 Probe Direction 5-2
5.4 Guidance Cues 5-2
5.4.1 Slide 5-3
5.4.2 Rotate 5-4
5.4.3 Tilt 5-4
5.4.4 Rock 5-5
5.4.5 Probe Guidance Interpretation 5-5
5.5 View Detection 5-7
5.6 Quality Bar 5-9
5.7 View Optimization 5-10
5.7.1 Depth 5-10
5.7.2 Gain 5-10

5.7.3 Patient Position 5-10
5.7.4 Respiration 5-10

CHAPTER 6 Scanning with UltraSight Guidance 6-1
6.1 Preliminary Preparations 6-1
6.2 Basic Workflow 6-2
6.3 Starting a New Study 6-3
6.4 Creating a Patient 6-3
6.5 Preparing the Exam 6-4
6.6 Preparing the Patient 6-4
6.7 Performing the Exam 6-4
6.8 Acquiring Loops 6-7
6.9 Continuing and Completing the Exam 6-7
6.10 Reviewing Acquired Loops 6-7

CHAPTER 7 Clinical Studies 7-1
7.1 Pivotal Study 7-1
7.1.1 Pre-Study Phase 7-1
7.1.2 Study Phase 7-1
7.2 Exams' Quality Evaluation By Expert Cardiologists 7-1
7.3 Primary Endpoint 7-2
7.4 Secondary Endpoints 7-3
7.5 Safety Profile 7-4
7.6 Human Factors Validation Study 7-4
7.7 Non-Clinical Testing 7-6

CHAPTER 8 Troubleshooting 8-1
8.1 Introduction 8-1
8.2 UltraSight Application Troubleshooting Guide 8-1
8.2.1 Heart Not Detected 8-1
8.2.2 Verify Position Depth and Gain 8-2

8.2.3 Low-Quality Indication without Guidance Cues ...8-2

CHAPTER 9 Appendices **9-1**

Appendix A. Windows and Views 9-1

A.1. Parasternal Window....................9-1

A.2. APICAL Window....................9-4

A.3. SUBCOSTAL Window 9-7

Appendix B. Supported Ultra-Sound Systems 9-9

B.1. Philips Lumify Ultrasound System....................9-9

List of Figures

Figure No. Description .... Page
Figure 3-1. The UltraSight interface 3-1
Figure 3-2. The 10 standard views 3-3
Figure 3-3. UltraSight AI Guidance layout 3-4
Figure 3-4. Screen zoom setting 3-8
Figure 4-1. UltraSight recording or casting permission 4-3
Figure 4-2. UltraSight AI Guidance overlay permission request 4-3
Figure 4-3. UltraSight notification permission 4-4
Figure 4-4. Main screen 4-4
Figure 4-5 UltraSight UI 4-5
Figure 4-6 Main menu 4-6
Figure 4-7. Settings 4-6
Figure 4-8. Admin password 4-7
Figure 4-9. Admin settings 4-7
Figure 4-10. About window 4-8
Figure 4-11. Exit dialog box 4-9
Figure 4-12. Cardiac views 4-10
Figure 4-13. Guidance button 4-11
Figure 4-14. Guidance On/Off 4-11
Figure 4-15. Static guidance 4-12
Figure 4-16. Tutorial button 4-13
Figure 4-17. Tutorial window 4-13
Figure 4-18. Dynamic real-time guidance 4-15
Figure 4-19. De-Brief mode 4-16
Figure 4-20 . Review Screen 4-17
Figure 4-21. Export button 4-18
Figure 4-22. Add folder 4-18

Figure 5-1. Static guide 5-1
Figure 5-2. Probe direction mark....5-2
Figure 5-3. Probe manipulations 5-2
Figure 5-4. Target and probe crosshairs 5-3
Figure 5-5. Sliding the probe 5-3
Figure 5-6. Rotate....5-4
Figure 5-7. Tilt....5-4
Figure 5-8. Rock 5-5
Figure 5-9. Hold position 5-8
Figure 5-10. Quality bar 5-9
Figure 6-1. Recommended workflow for optimal scan views 6-1
Figure 6-2. Opening screen 6-3
Figure 6-3. Acoustic Window instruction 6-5
Figure 6-4. Tilt instruction 6-6
Figure 6-5. Quality bar with a high score 6-6
Figure 8-1. Heart not detected 8-1
Figure 9-1. PLAX view....9-1
Figure 9-2. PSAX-AV view 9-2
Figure 9-3. PSAX-MV view 9-2
Figure 9-4. PSAX-PM view 9-3
Figure 9-5. APICAL 4C view....9-4
Figure 9-6. APICAL 3C view....9-5
Figure 9-7. APICAL 2C view....9-5
Figure 9-8. APICAL 5C view....9-6
Figure 9-9. SUBCOSTAL SC-4C 9-7
Figure 9-10. SUBCOSTAL IVC-4C 9-8

List of Tables

Table 1. Primary endpoints results 7-2
Table 2. Secondary endpoints results 7-3
Table 3. Secondary endpoints results (of the 10 views) 7-3
Table 4: Data used for Algorithm Development and Performance Testing 7-6

CHAPTER 1

Introduction

1.1 About this Manual

This manual has been prepared to aid the user in using the UltraSight AI Guidance.

It provides information on configuring and using the UltraSight AI Guidance software to capture diagnostic-quality ultrasound video clips of the heart.

Please read this operator's manual in its entirety before using the UltraSight AI Guidance software.

Note
This manual describes how to use the UltraSight AI Guidance when installed on a compatible Ultrasound System. The instructions describe the most commonly used applications. Some functions described in this manual may look different on other compatible Ultrasound Systems, but the operation is the same.

1.2 Conventions

The Conventions section explains the basic symbols used in this manual. Three types of special messages may appear in this manual:

Symbol	Description
	**Warning**: Indicates a precaution to avoid possible injury or death.
	**Caution**: Indicates a condition that may lead to damage of equipment, lower quality of use, or loss of information.
	**Note**: Indicates supplemental information and provides a tip on how best to use it.
	**Reference**: Refer to the Ultrasound system's user manual (Philips Lumify).

1.3 Warnings

The following is important information regarding patients, operators, and equipment safety.

Warning

MRI Safety Information

The UltraSight AI Guidance is MR Unsafe. Keep the UltraSight AI Guidance software outside the MRI Scanner room.

Warning

Ultrasound-system hazards

General ultrasound-system hazards not specific to UltraSight AI Guidance are described in the Ultrasound System manuals.

Read the Ultrasound System manual thoroughly before using it with the UltraSight AI Guidance software.

Warning

Image quality and diagnosis

Product users are responsible for image quality and diagnosis. The video clips acquired using UltraSight AI Guidance assistance are to be interpreted only by qualified medical professionals. The qualified medical professional must inspect the data used for analysis and diagnosis and ensure that the data is sufficient and appropriate in anatomical correctness and spatial and temporal resolution for the measurement being employed.

Warning

Probe compatibility

UltraSight AI Guidance operates with the cardiac ultrasound probe S-4 linear phased array probe used by the compatible Lumify Ultrasound System (called a “transducer” in the manual).

This is a warning for the Ultrasound System, so do not operate the UltraSight AI Guidance with any other probe.

Warning

UltraSight AI Guidance accuracy, use, and image saving

UltraSight AI Guidance provides real-time guidance during cardiac ultrasound (echocardiographic) examinations for 10 echocardiographic views.

The accuracy of UltraSight AI Guidance in classifying correct echocardiographic views and estimating image quality has been verified and validated, but individual patient variations may introduce errors. As many echocardiographic views are similar to other views, errors may occur. Therefore, it is important to review saved video clips independently using experienced clinical judgment prior to diagnosis.

Making a diagnosis based solely on UltraSight AI Guidance without applying clinical judgment regarding view correctness and quality is not recommended.

Warning

Prescriptive Guidance instructions

UltraSight AI Guidance provides prescriptive guidance that suggests how to manipulate the probe to capture the desired images. These suggestions have been verified and validated, but individual patient variations may cause the instructions to perform better in some patients than others.

Pay attention to the quality meter while manipulating the probe and save the video clip only when the quality meter indicates the probe is in a good position.

Warning

ALARA considerations

When conducting ultrasound studies, follow the ALARA principle: expose the patient to ultrasound energy at a level that is "As Low As Reasonably Achievable."

Please read the Lumify Ultrasound System User Guide for important information about acoustic output and ALARA.

1.4 Cautions

Below is important information for preventing the loss of patient or product data or preventing damage to the UltraSight AI Guidance device or environment.

Caution

Cybersecurity precautions and practices

Malware, computer viruses, ransomware, and other cybersecurity threats are an increasing concern in healthcare IT systems.

Please refer to the Lumify Ultrasound System User Guide for important information about cybersecurity provisions and management. For information and guidance on implementing proper cybersecurity in the healthcare IT environment, see "Health IT Privacy and Security Resources for Providers" at https://www.healthit.gov/topic/privacy-security-and-hipaa/ for information and guidance on implementing proper cybersecurity in the healthcare IT environment.

Caution

Product compatibility

UltraSight AI Guidance software is a software accessory that operates only with the compatible ultrasound system Philips Lumify S4-1 ultrasound transducer. It is not designed to operate with any other ultrasound systems. Do not attempt to operate UltraSight AI Guidance with other ultrasound systems.

Do not use your system in combination with other products or components unless UltraSight expressly recognizes those other products or components as compatible. For information about such products and components, contact your UltraSight representative.

Changes and additions to the system shall be made only by UltraSight Ltd., or by third parties expressly authorized by UltraSight. Such changes and additions must comply with all applicable laws and regulations that have the force of law within the jurisdictions concerned and with best engineering practices.

Caution

Intended audience for the UltraSight AI Guidance

UltraSight AI Guidance must be operated by or under the direction and supervision of a licensed physician.

1.5 Intended Purpose

The UltraSight AI Guidance software is intended to assist medical professionals (not including expert sonographers) in performing cardiac ultrasound scans.

UltraSight AI Guidance software is an accessory to compatible general-purpose diagnostic ultrasound systems.

1.6 Intended User

This manual is intended to be used by medical professionals (not including expert sonographers) who have received appropriate training on ultrasound basics and training on using the UltraSight AI Guidance software, provided by either UltraSight or by a trained clinician while using UltraSight training materials.

1.7 Indication for Use

The UltraSight AI Guidance software is indicated for use in two-dimensional transthoracic echocardiography (2D-TTE) for adult patients, specifically in the acquisition of the following standard views:

- Parasternal Long-Axis (PLAX)
- Parasternal Short-Axis at the Aortic Valve (PSAX-AV)
- Parasternal Short-Axis at the Mitral Valve (PSAX-MV)
- Parasternal Short-Axis at the Papillary Muscle (PSAX-PM)
- Apical 4-Chamber (AP4)
- Apical 5-Chamber (AP5)
- Apical 2-Chamber (AP2)
- Apical 3-Chamber (AP3)
- Subcostal 4-Chamber (SC-4C)
- Subcostal Inferior Vena Cava (SC-IVC)

The UltraSight AI Guidance is not intended to be used by Sonographers.

1.8 Contraindications

There are no contraindications for using the UltraSight AI Guidance software.

1.9 Clinical Benefits and Precautions

1.9.1 Clinical Benefit

UltraSight AI Guidance provides a means for any medical professional (not including expert sonographers) to perform a cardiac ultrasound for diagnosis and eventually treatment after limited and short training.

1.9.2 Precautions Regarding Specific Patient Types

UltraSight AI Guidance was thoroughly tested for use only on adults. It is not recommended for use and may cause false guidance cues and quality indications in patients with the following conditions:

- Congenital heart disease with complex anatomy such as:
    - Single ventricle anatomy
    - Tricuspid Atresia
    - Pulmonary Atresia
    - Double Outlet Right Ventricle
    - Any complex congenital heart anatomy
- Abnormal position of the heart and great vessels such as:
    - Dextrocardia
    - Dextroversion
    - Dextroposition
- Permanent long term, mechanical support devices such as LVAD/RVAD.

1.10 Abbreviations and Acronyms

AI	Artificial Intelligence
ASE	American Society of Echocardiography
CFR	Code of Federal Regulations
GNN	Guidance Neural Network
HUD	Handheld Ultrasound Devices
ICS	Intercostal space
POCUS	Point of Care Ultrasound
PSB	Parasternal Border
RN	Registered nurse
TTE	Transthoracic echocardiography

CHAPTER 2

Safety Considerations

2.1 Introduction

This chapter describes general safety issues regarding the use of the UltraSight AI Guidance software on compatible diagnostic ultrasound systems.

Warning

Before using the UltraSight AI Guidance, read this manual and strictly observe all warnings and cautions.

Warning

Legal considerations

The installation, use, and operation of this product are subject to the law in the jurisdictions in which the product is used. Install, use, and operate UltraSight AI Guidance only in a manner that does not conflict with applicable laws or regulations. Using the product for purposes other than those intended and expressly stated by UltraSight Ltd. and incorrect use or operation may relieve UltraSight or its agents from all or some responsibility for resultant noncompliance, damage, or injury.

Caution

Federal (United States) law restricts this device to sale by or on the order of a physician.

Note

Any serious incident related to the device should be reported to the manufacturer and the competent authority of the Member State where the user and/or patient is established.

2.2 Equipment Classification and Compliance

Following are the Ultrasound Systems classification and compliance. For more details, check the Ultrasound System manuals.

2.2.1 Classification

- Device with transducers: Internally powered ME equipment. Transducers: Type BF applied parts, IP47
- Ordinary Equipment/Continuous Operation
- Non-AP/APG

2.2.2 Electromechanical Safety Standards Met

The transducers and software comply with IEC 60601-1 Medical Electrical Equipment, General Requirements for Safety, including all applicable collateral and particular standards, and all applicable deviations. System users are responsible for ensuring that the chosen device complies with the law in the jurisdiction in which the product is used.

2.2.3 Compliance

The Ultrasound Systems compatible with the UltraSight AI Guidance software comply with relevant international and national standards and laws.

The equipment manufacturer will supply compliance information upon request.

2.3 Biological Safety

Reference

Information about biological safety and a discussion of the cautious use of the system is provided in the Ultrasound System manuals.

A list of precautions related to biological safety is indicated in the manuals; observe these precautions when using the system.

CHAPTER 3

System Description

3.1 Introduction

The UltraSight AI Guidance software pairs with Ultrasound Systems used for conducting cardiac ultrasound at the point of care.

Its AI technology assists medical professionals by providing real-time on-screen guidance and positioning quality assessment.

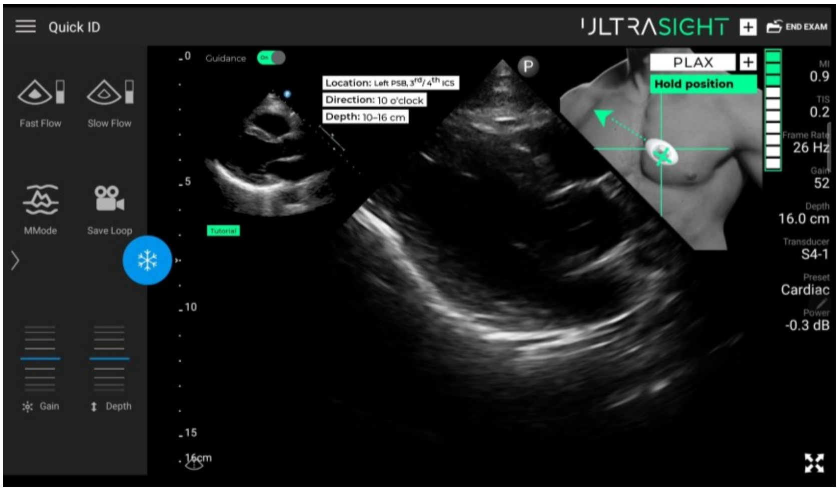

Figure 3-1. The UltraSight interface

3.1.1 Medical Background

Echocardiography is the most widely used imaging modality in cardiology and is the foundation for cardiac diagnostic testing, allowing for direct identification and management of various conditions. It permits rapid assessment of cardiac size, structure, function, and hemodynamics.

Ultrasound is more portable and less expensive compared with other imaging modalities (computed tomography, magnetic resonance imaging, nuclear perfusion imaging), and unlike methods that expose patients to radiation, there are no known adverse effects of ultrasound used at diagnostic imaging intensities, allowing safe, serial evaluation of patients.

Ultrasound images can be evaluated in real-time, which allows rapid diagnostic interpretation in a wide variety of clinical settings, such as the outpatient clinic, inpatient ward, critical care unit, emergency department, operating room, remote clinic, and cardiac catheterization laboratory.

Despite the above benefits, one of the main challenges of echocardiography is that it is a user-dependent method that requires intensive training to acquire quality sufficient ultrasound images of the heart and achieve accurate interpretation of the acquired data.

To address the need to assist medical professionals in acquiring cardiac ultrasound images, UltraSight has developed the UltraSight AI Guidance software, a machine learning-based software application, as a system for guiding healthcare providers without experience in echocardiography to acquire high-quality ultrasound imagery of the heart.

3.2 Device Description

3.2.1 General

UltraSight AI Guidance software is a machine learning-based, downloadable mobile application that utilizes artificial intelligence (AI) to provide real-time adaptive guidance of transducer positioning and orientation to help intended users acquire diagnostic-quality echocardiographic views of the heart.

The system provides guidance for the 10 most common cardiac views via three acoustic windows (Parasternal, Apical, and Subcoastal).

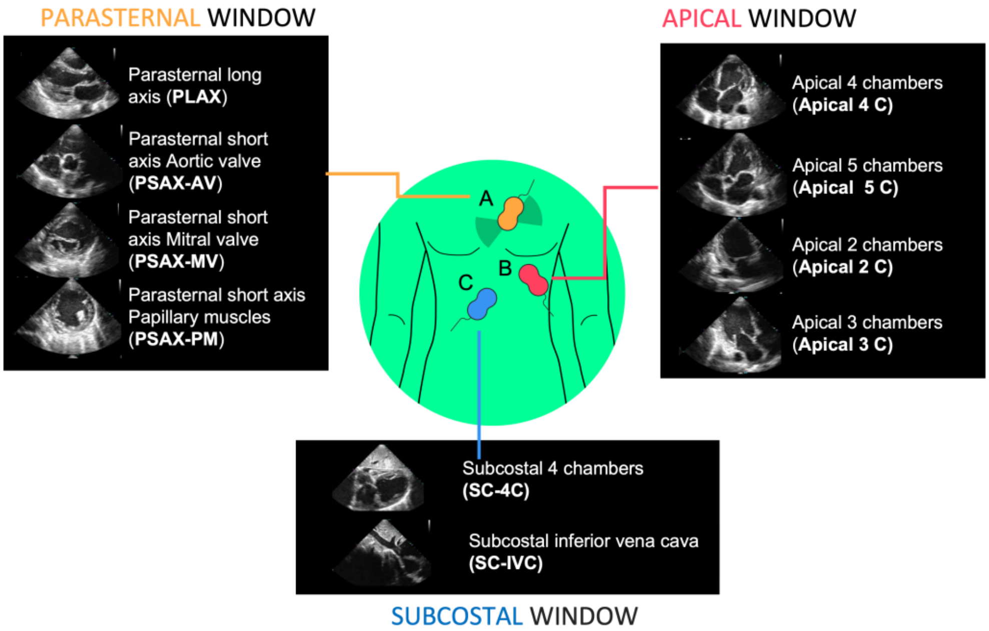

Figure 3-2. The 10 standard views

3.2.2 Application Description

UltraSight AI Guidance is a software-only device that is installed on and interfaces with the Philips Lumify transportable handheld ultrasound system.

Figure 3-3 depicts the main building blocks of the UltraSight application clinical use.

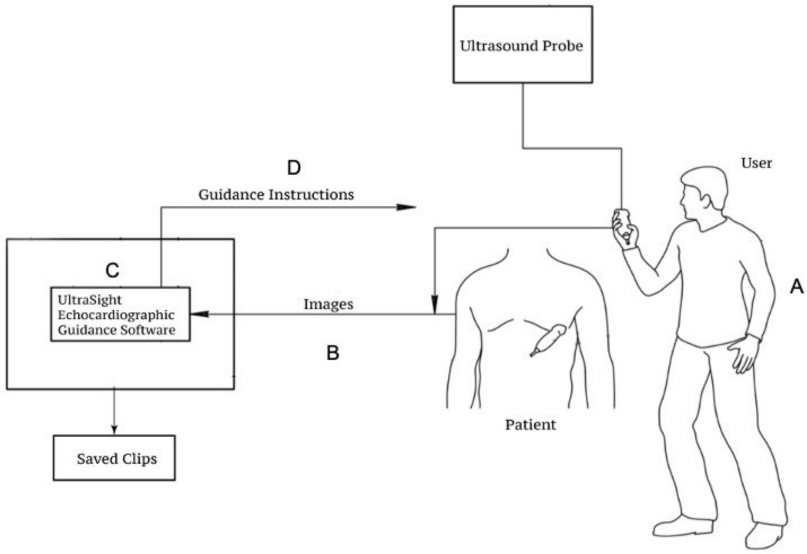

Figure 3-3. UltraSight AI Guidance layout

1. A user (A) scans a patient using an approved U/S probe.
2. The Ultrasound clips are used as an input to the UltraSight software (B) that is installed and running on a tablet (C). This is the same tablet the ultrasound software runs on.
3. Based on the input images, the UltraSight software guides the user (D) on where to place the transducer and how to manipulate it to acquire an optimal view.
4. The clips are saved on the Ultrasound tablet with the same functionality as in a standard echocardiography exam, using Philips Lumify transportable handheld ultrasound system's User Interface.

3.2.3 About the UltraSight AI Guidance Software

UltraSight AI Guidance software is a standalone Android application that is installed on an off-the-shelf Samsung Tab S6/ S7/ S7-5G/ S8/ S9 or S9 Plus tablet used by the Ultrasound System. The application uses Android's screen capturing mechanism to receive a stream of images from the Philips Lumify ultrasound application and analyze them in real-time in a Deep Learning-based machine learning core.

The analysis results are displayed as guidance cues and image quality assessment indications, which are overlaid on top of the Philips Lumify user interface in a manner that does not interfere with the ultrasound image and other information shown by the Philips Lumify application.

The system stops the guidance when the UltraSight AI Guidance identifies that scanning is not in progress. The system also understands whether Lumify is using right-hand or left-hand scanning and whether the Philips Lumify is inverted in the display.

When the UltraSight application is installed and in use, all the features and capabilities of the ultrasound system application (Philips Lumify Ultrasound System) are preserved. They can be operated regularly, with no interference from the ongoing operation of the UltraSight AI Guidance software.

3.2.3.1 Software Limitations

The UltraSight AI Guidance software operates in parallel to the Phillips Lumify software running on the handheld device and is active and tested only in the following mode:

- B-Mode 2D Landscape (regular and expanded mode)

> *Caution*
>
> UltraSight software does not provide guidance for the other ultrasound modes of the Phillips Lumify software (Doppler, M-Mode, Freeze, etc.), including Portrait mode view (regular and expended) and ZOOM feature in all B-Mode 2D functions.

3.2.3.2 Software Features

The UltraSight AI Guidance user interface supports the acquisition of 10 views via three acoustic windows (Apical, Parasternal, and Subcostal).
Each view has a tutorial window that guides the user on the suitable depth, where to place the probe on the patient, and the direction of the Lumify probe marker (Figure 5-2) for acquiring the view at the required acoustic window.

The Guidance screen has three features that assist the user with acquiring the echocardiography views:

- Navigation Probe Guidance – graphic cues that provide guidance on where to place the probe and how to manipulate it to reach the desired view (slide, tilt, rock, and rotation).
- Quality Bar – The Quality Bar indicates the estimated quality of the current view in reference to an optimal view of the specific cardiac view.
- View detection – The software differentiate between three states, and the display adjusts accordingly:
    - No heart - heart is not visible, the user is expected to first find the acoustic window where some heart tissue is visible and then follow the navigation instructions.
    - Navigate - the quality bar is below 8 bars, the heart is visible, the user should watch the guidance cues and move the probe accordingly.
    - Hold position - the quality bar has reached 8 bars (or more), the tail of the probe turns green, and the user should record a clip.

3.2.4 System Requirements

The UltraSight AI Guidance software was tested and verified with the Philips Lumify ultrasound system, CE Marked, and FDA-cleared under K162549.

The tests were performed in the following configuration:

- Philips Lumify S4-1ultrasound transducer, Lumify Power Module (LPM) Philips, Philips part number 453561998451.
- Philips Lumify application – Philips Lumify, software number/version: 4.0 453562133201 21-11-16 or 4.0.1 453562163981 22-09-15.

Caution

The Lumify Application must be updated only to versions supported by the UltraSight AI Guidance application.

3.2.4.1 Tablet Requirements

Following are the supported tablets and their specifications.

- Supported tablet devices:
  - Samsung Galaxy Tab S6 (SM-T860 & SM-T865).
  - Samsung Galaxy Tab S7 (SM-T870, SM-T875 & SM-T878).
  - Samsung Galaxy Tab S8 (SM-X700).
  - Samsung Galaxy Tab S9 (SM-X710).
  - Samsung Galaxy Tab S9 Plus (SM-X810).
- Tablet software:
  - Tablet OS – Android version 11 and above.
- Memory Storage:
  - Minimum 6GB of RAM memory.
  - Minimum storage space for installation: 1GB.

> *Note*
>
> The UltraSight AI Guidance application uses only the device's processing power and does not require internet connectivity (except for the registration process).

3.2.4.2 Tablet Settings

The screen zoom of Android tablets must be set to their default level.

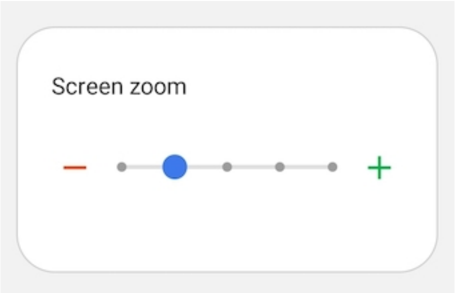

Figure 3-4. Screen zoom setting

3.3 Device Labels

Symbol	Definition
MD	The medical device symbol .
CE	The CE Marking symbol.
Rx only	Federal (United States) law restricts this product to sale by or on the order of a physician.
UDI	The unique device identification number symbol.
MR	MR unsafe symbol.
EC REP Donawa Lifescience Piazza Albania, 10 00153 Rome, Italy	The symbol for Authorised Representative.
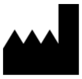	The product manufacturer, including address.

CHAPTER 4

The UltraSight Application

4.1 Installing the Application

Downloading and installing the UltraSight AI Guidance App requires an internet connection.

> *Note*
>
> Using the UltraSight AI Guidance software application requires installing the Lumify Ultrasound System application.

To connect the tablet to the internet:

1. Verify that the "Lumify Ultrasound System" application is installed on the tablet.
2. Press the tablet **Settings** icon and select **Connections**.
3. Ensure the **Wi-Fi** sliding button is switched on and press the **Wi-Fi** field. A list of the available Wi-Fi networks in the range is displayed.
4. Select your network. If the network is secured, enter the password and tap **Connect**.

To download the UltraSight App:

1. Open **Google Play Store** on the tablet.
2. Search for the **UltraSight™** App and its logo.
   The App is free-of-charge.
3. Tap **Install** to install the UltraSight™ App. The App is installed automatically after downloading from the Google Play Store.

> *Note*
>
> Software updates shall be available on Google Play.

4.2 Registration and Entitlement

Using the UltraSight AI Guidance application requires registering an account with UltraSight.

Once UltraSight approves the account, each account receives an activation key per acquired license that needs to be activated on the tablet device.

While the tablet is connected to the internet, the activation key must be inserted in the activation key dialog in the UltraSight AI Guidance application to enable the functionality of the UltraSight AI Guidance application.

> *Note*
>
> The activation key has an expiration date that requires renewal for the application to continue working.

4.3 Launching the UltraSight Application

1. Connect a compatible Ultrasound Probe to the tablet for the application to fully function and display the Cardiac scan.

> *Note*
>
> If the cardiac probe is not connected to the tablet USB-C port, the Cardiac scan mode will not be available.

2. Tap the **UltraSight** application icon on your device.

   The following notification message appears.

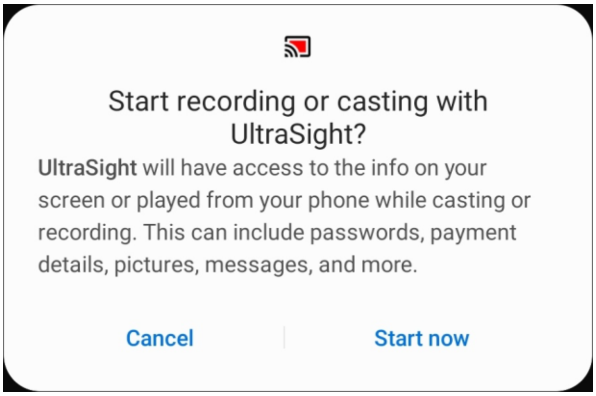

Figure 4-1. UltraSight recording or casting permission

3. Press **Start now**.

   The UltraSight application receives the necessary permissions to start using the UltraSight AI Guidance.

4. Notification for permission to display over other apps.

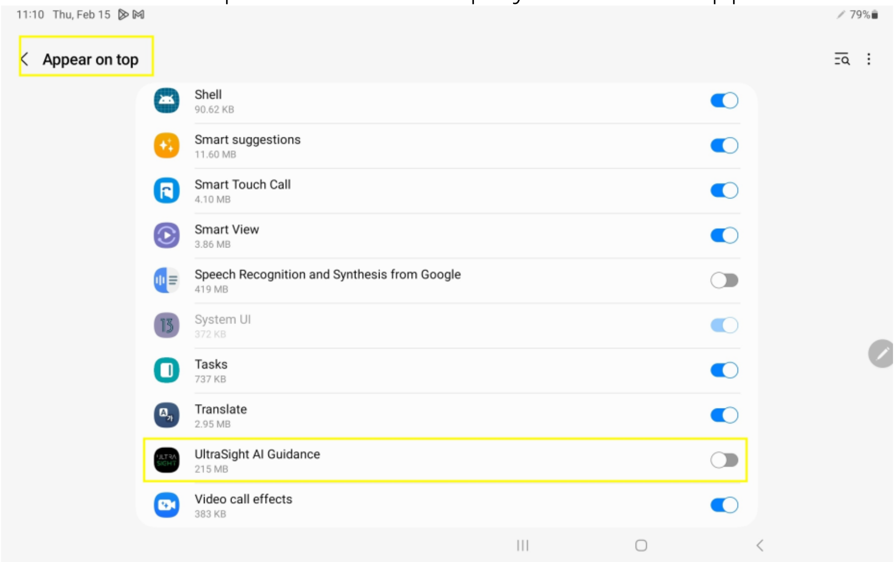

Figure 4-2. UltraSight AI Guidance overlay permission request

5. Tap on UltraSight AI Guidance to enable.

6. Request for UltraSight AI Guidance to enable notifications.

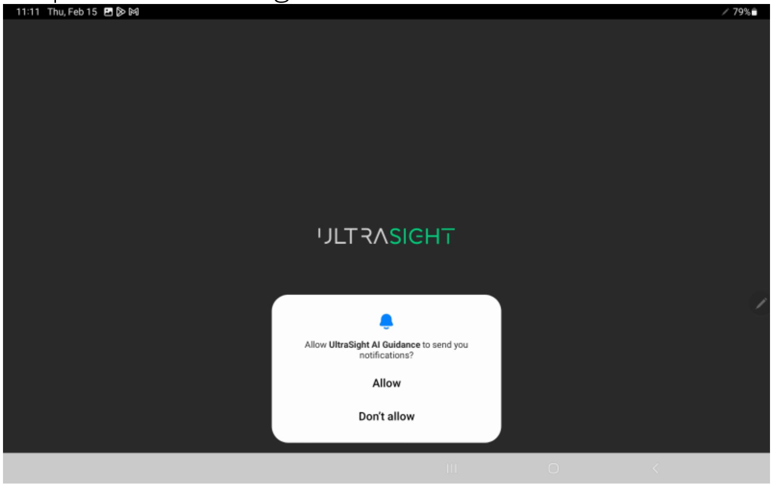

Figure 4-3. UltraSight notification permission

7. Press “Allow”.

> *Notes*
>
> Launching the UltraSight AI Guidance application opens the basic U/S operating application automatically, if it is not already open, with the UltraSight software overlay.
>
> The UltraSight AI Guidance user interface appears differently depending on the hosting Ultrasound device.

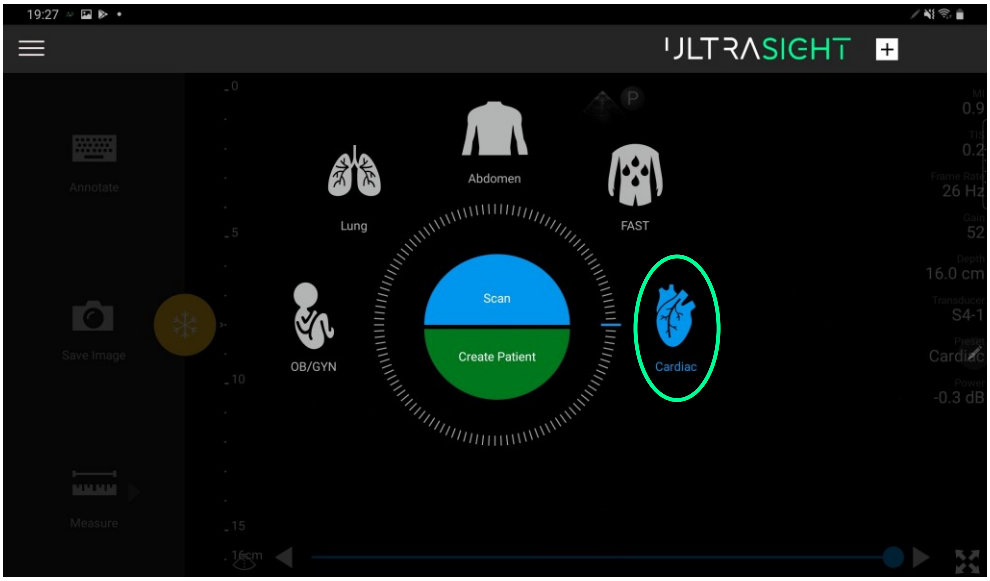

Figure 4-4. Main screen

4.4 The UltraSight Application

The UltraSight application has the following displays and functions:

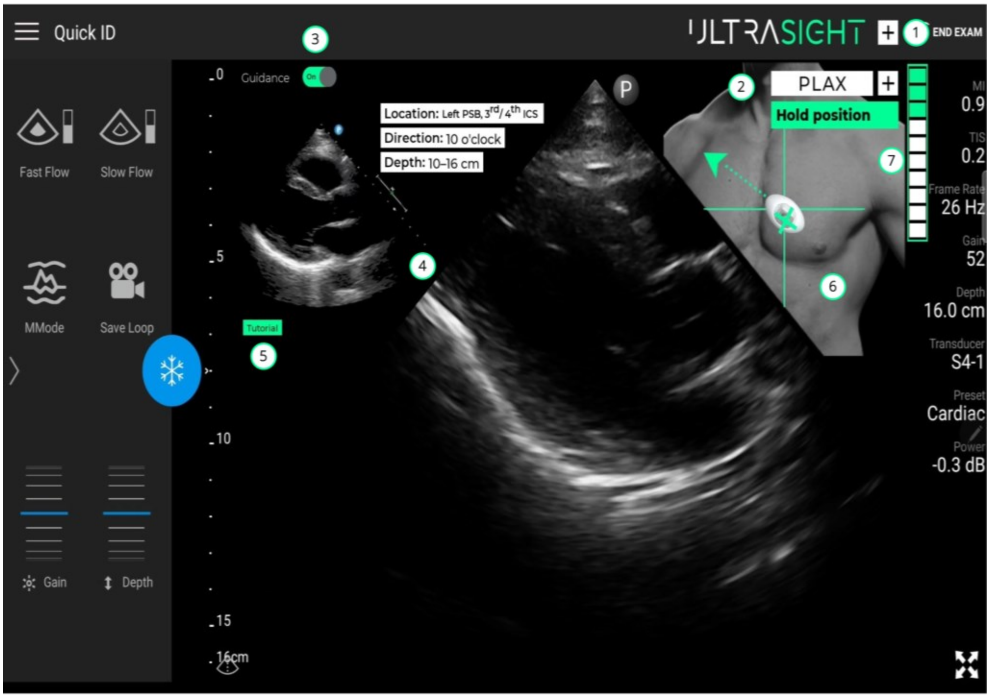

**1**	Main menu	**5**	Tutorial Button
**2**	View selection menu	**6**	Active real-time guidance
**3**	Guidance master switch	**7**	Quality bar
**4**	Static Guidance		

Figure 4-5 UltraSight UI

4.4.1 Main Menu

Pressing the main menu [+] icon opens the UltraSight main menu.

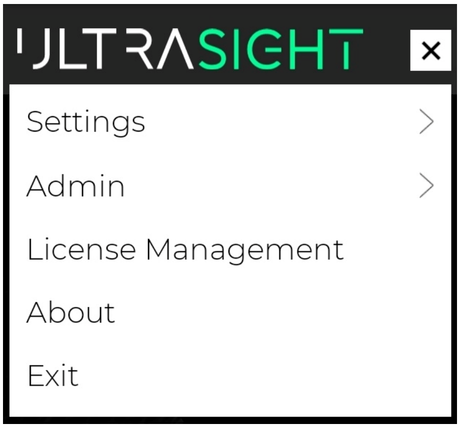

Figure 4-6 Main menu

4.4.1.1 Settings

The Settings menu is used to set the default view and what is displayed on the UltraSight user interface by default.

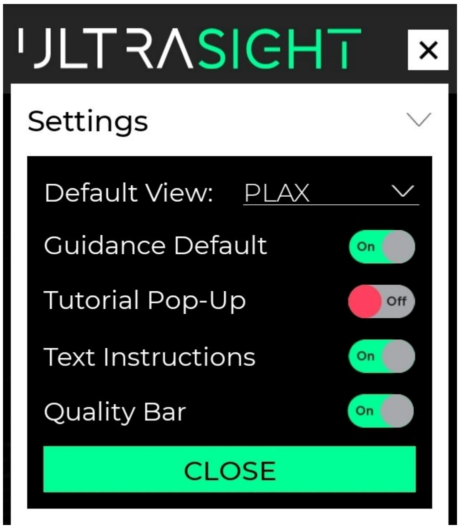

Figure 4-7. Settings

- **Default View** – enables selecting the default cardiac view when starting an exam with the aid of the UltraSight AI Guidance app.
- **Guidance Default** – If set to “On”, the Guidance Instructions automatically appear when opening the UltraSight AI Guidance application.

- **Tutorial Pop-Up** – If set to "On", the Tutorial window appears automatically each time a cardiac view is selected.
- **Text Instructions** – If set to "Off", the Popup Notifications do not appear in the Dynamic Guidance section during a scan.
- **Quality Bar** – set to On/Off to present or hide the Quality Bar.

4.4.1.2 Admin

Entering the UltraSight software Admin mode requires entering a password.

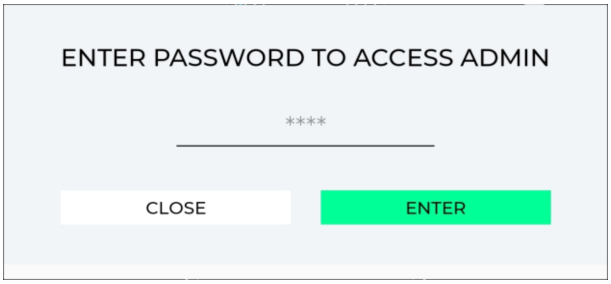

Figure 4-8. Admin password

In Admin mode, the **De-Brief Mode** feature can be activated.

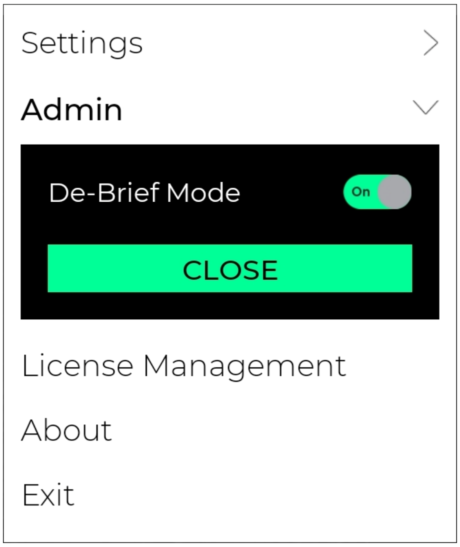

Figure 4-9. Admin settings

For details on the **De-Brief Mode**, see section 4.4.7.

4.4.1.3 About

Tapping ABOUT displays the following data window.

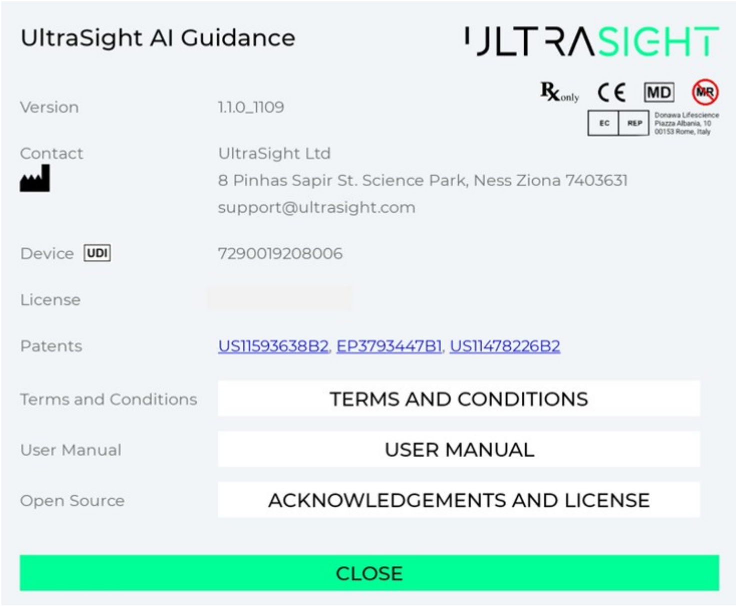

Figure 4-10. About window

- Software logo
- Software name
- Software version
- UltraSight contact information
- Device UDI number
- License number
- Patents
- Terms and Conditions – Taping opens the “Terms and Conditions” in the UltraSight AI Guidance app.
- User Manual – Taping opens the UltraSight AI Guidance app “User Manual” on the browser installed on the tablet.
- Open Source – Taping opens the UltraSight AI Guidance app “Acknowledgements and License”.
- Device labels.
- Close – Tap to close the About window.

4.4.1.4 Exit

Pressing **Exit** displays the following dialog box.

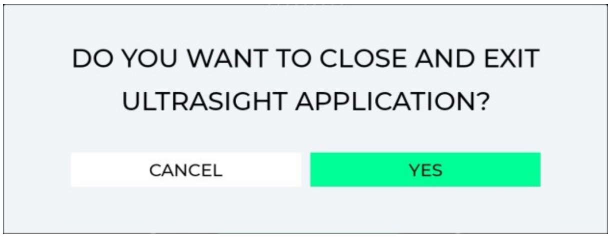

Figure 4-11. Exit dialog box

- Select **Cancel** to remain in the UltraSight AI Guidance application.
- Select **Yes** to exit the UltraSight AI Guidance app.

Note

When exiting the UltraSight AI Guidance app, the Lumify application continues to operate.

4.4.2 Views Menu

The UltraSight guidance system provides navigation assistance for the acquisition of ten cardiac views: Parasternal Long-Axis (PLAX), Parasternal Short-Axis at the Aortic Valve (PSAX-AV), Parasternal Short-Axis at the Mitral Valve (PSAX-MV), Parasternal Short-Axis at the Papillary Muscle (PSAX-PM), Apical 4-Chamber (AP4), Apical 5-Chamber (AP5), Apical 2-Chamber (AP2), Apical 3-Chamber (AP3), Subcostal 4-Chamber (SC-4C), Subcostal Inferior Vena Cava (SC-IVC).

> *Note*
>
> The default view is set in the Settings menu (see section 4.4.1.1).

To select a view:

1. Tap the + on the views menu to display the 10 Cardiac views.

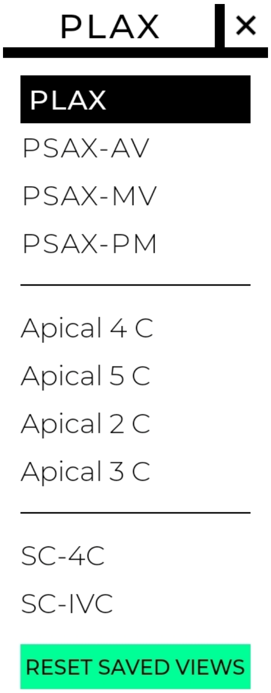

Figure 4-12. Cardiac views

2. Select the required view.

   The dropdown menu closes, its header changes to the selected view, and if the **Guidance switch** is set to **On** (see section 4.4.3), the static and dynamic guidance instructions appear.

4.4.3 Guidance Switch

Enabling guidance is performed by the **Guidance** On/Off button.

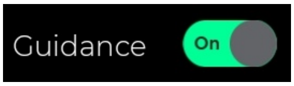

Figure 4-13. Guidance button

The default status of the Guidance button is set in the application Settings menu (see section 4.4.1.1 above).

<u>Guidance On</u>

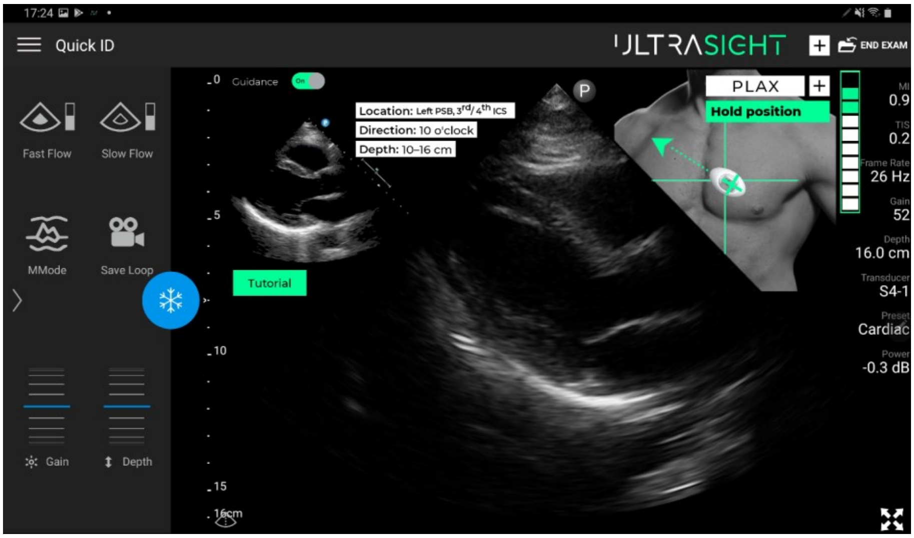

<u>Guidance Off</u>

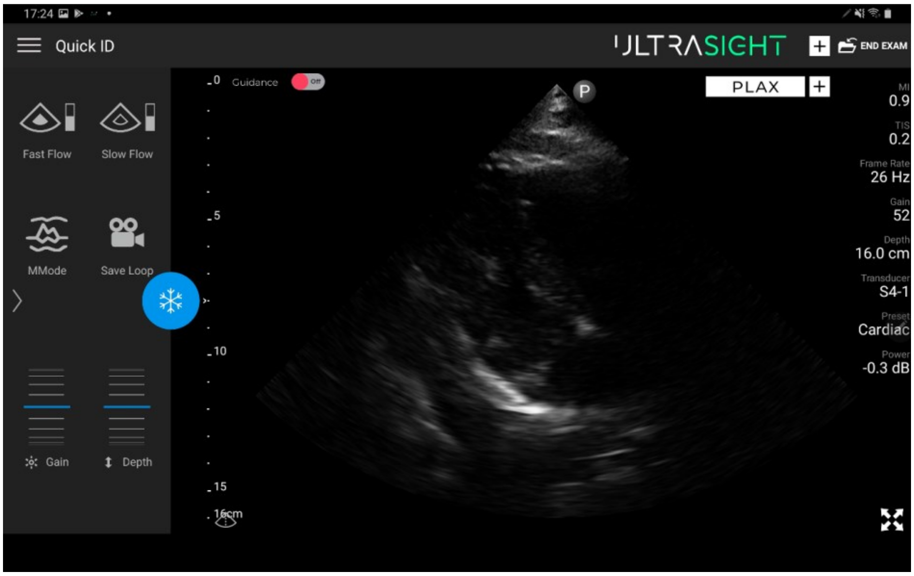

Figure 4-14. Guidance On/Off

4.4.4 Static Guidance

The Static Guidance section is displayed on the left side of the user interface and provides basic information that can assist with initially positioning the probe on the patient's chest.

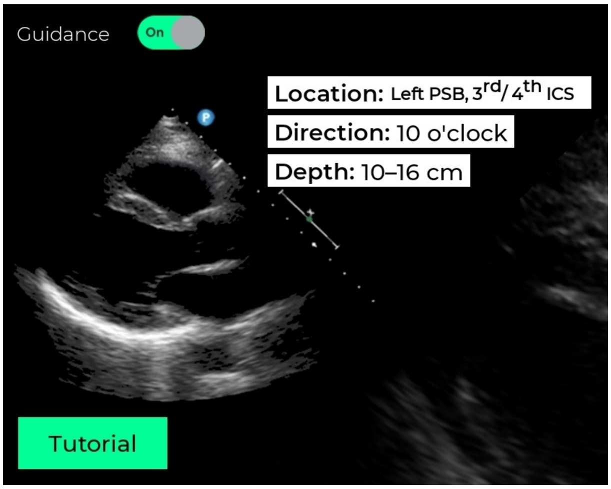

Figure 4-15. Static guidance

The Static Guidance instructions provide the following information:

- **Location** – the initial anatomical location to place the probe on the patient's chest (e.g., Left parasternal border, between the 3rd and 4th intercostal space).
- **Direction** – the initially recommended direction of the probe's pointer for the selected cardiac view (e.g., 10 o'clock).
- **Depth** – the initially recommended depth to start requiring the cardiac view (e.g., 10-16 cm).
  Note: Depth is set through the Lumify application.

In addition to the static guidance, an image of the selected cardiac view is displayed, giving a better visual for the endpoint of the required view.

At the bottom of the static guidance section is a **Tutorial** button. Pressing this button opens an extended window with additional information for the acquiring process of the selected cardiac view (see details in the following section 4.4.5).

4.4.5 Tutorial

The **Tutorial** button is located beneath the static guidance section.

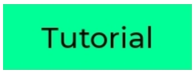

Figure 4-16. Tutorial button

Taping the Tutorial button opens the Tutorial window with the following information:

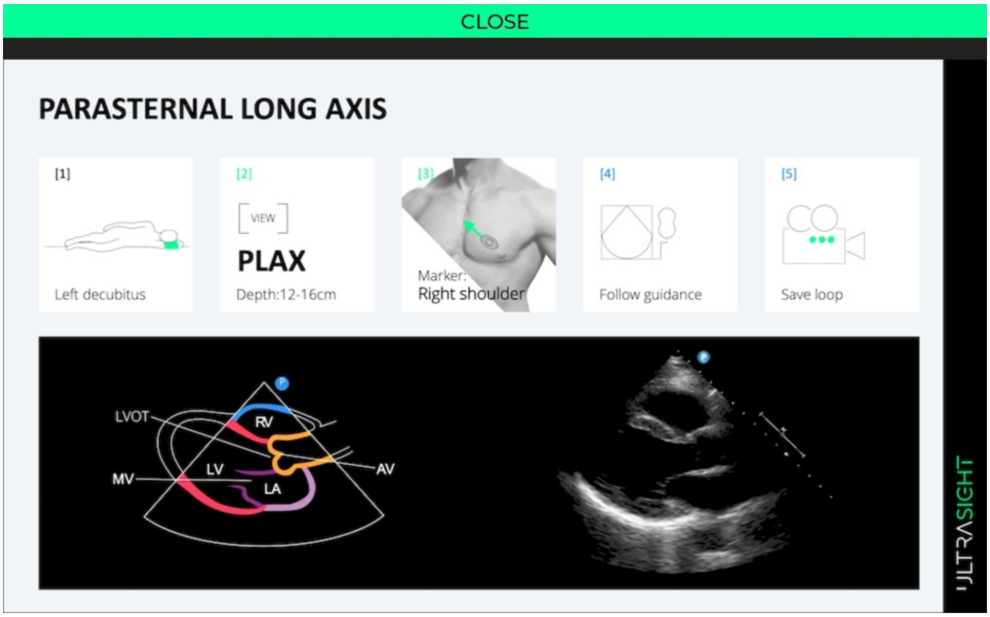

Figure 4-17. Tutorial window

- Image acquisition workflow (Figure 4-17):
  - [1] – The initial recommended position to locate the patient.
  - [2] – The initially recommended depth to start acquiring the cardiac window (e.g., 10-16 cm).
  - [3] – The initial recommended direction of the probe's pointer at the required cardiac view (e.g., Right Shoulder).
  - [4] & [5] – Instructions for completing the cardiac acquisition process ("Follow Guidance" and "Save Loop", respectively).
- Endpoint images – giving a basic understanding regarding the final cardiac view that needs to be acquired.

To close the Tutorial window, press the **Close** button on the window’s upper side.

4.4.6 Dynamic Real-Time Guidance

During the scanning process, dynamic real-time guidance is displayed, with guidance to reach and acquire the best view.

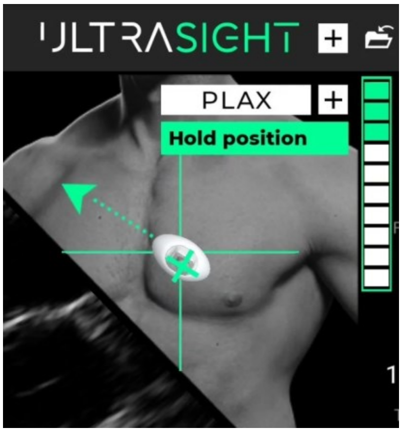

Figure 4-18. Dynamic real-time guidance

The Guidance instructions consist of the following information:

- **Probe Guidance** – composed of a 3D probe display that shows orientation guidance cues (rotations, tilts and rocks); a dashed line that indicates the direction of the probe marker; a fixed green arrowhead that indicates the target rotation angle; and a cross that shows slide guidance cues (slides in x and y directions).
- **Qualiy bar**– The quality bar is a score for image diagnosability. The quality bar consists of 10 bars (can be empty as well), the seven bottom ones are white, the top three are green. One or more green bars (8-10 bars) represent a high quality image, along with the tail of the probe that turns green and a text message "Hold position" that is indicated.
- **Text Instructions** – located at the upper side of the Active Guidance section, appear when a specific instruction is recommended (e.g., Adjust Depth, Hold position).

Refer to Chapter 5 for a detailed description of the guidance cues and quality bar. Refer to Chapter 6 for a detailed description on how to use the guidance cues and quality bar while performing an exam.

4.4.7 De-Brief Mode

<u>Note:</u> The De-Brief Mode can be activated only by an Administrator or Clinical specialist to record data for software performance and accuracy analysis and sent to UltraSight Customer service if needed.

De-Brief Mode allows recording the examination with the guidance area while using the UltraSight AI Guidance app. It can be used to analyze the UltraSight AI Guidance performance and accuracy.

After activating the De-Brief mode in the **Admin** menu (see section 4.4.1.2), two icons appear at the bottom of the screen, enabling the user to record the entire screen and review the recordings.

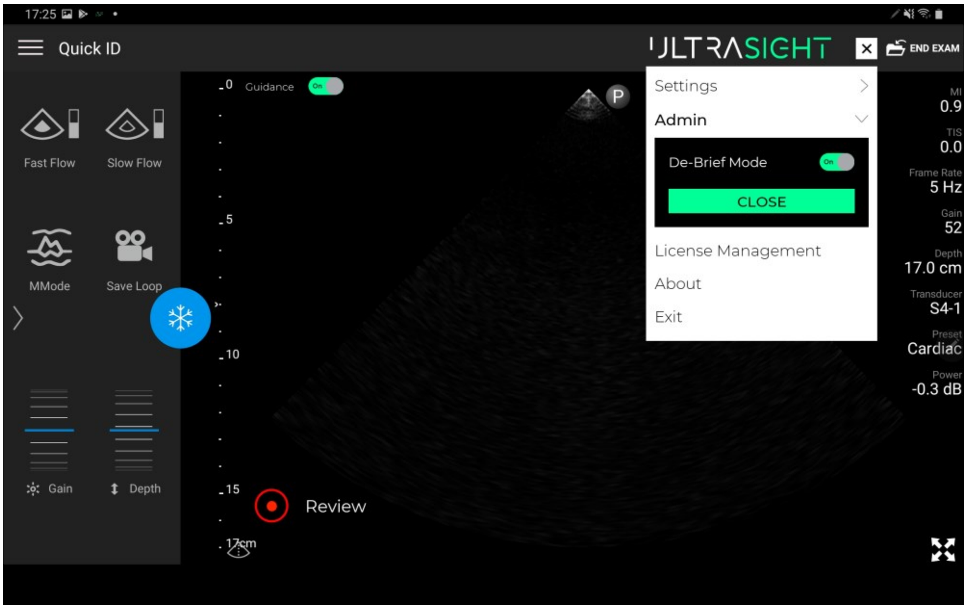

Figure 4-19. De-Brief mode

- **Screen Record** – pressing this button initiates a recording of the screen. To stop the recording, press the button a second time. The maximum screen recording length is 90 seconds.
- **Review** – pressing the title opens a separate window to review the recorded screen videos.

4.4.7.1 Review Screen

This Review Screen allows reviewing and exporting the recorded videos.

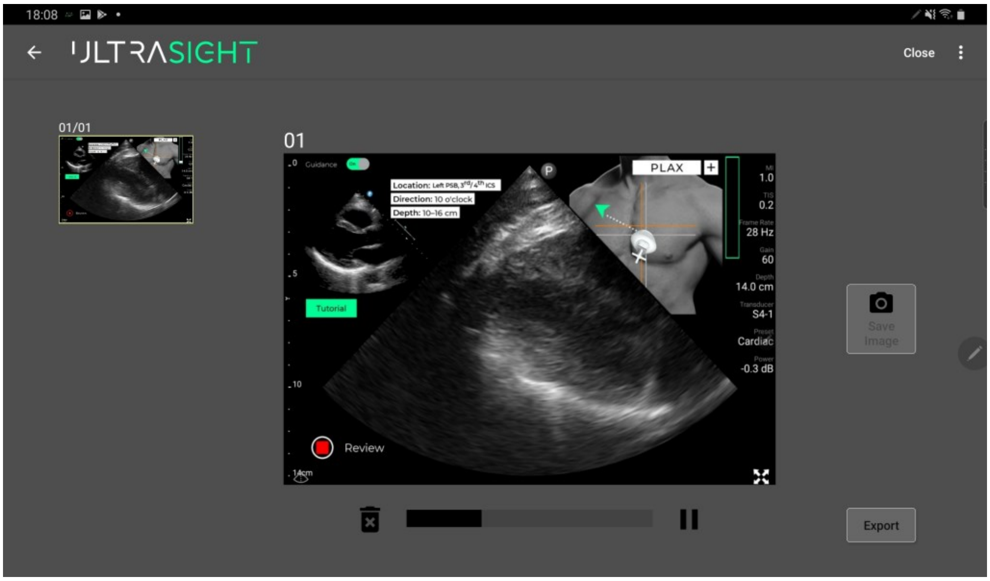

Figure 4-20 . Review Screen

The Review Screen window has the following features:

- **Video Selection** – a section that contains all the recorded videos. When selecting a video, it automatically starts playing at the center of the screen.
- **Operation Panel** – a section that allows pausing/playing the video and deleting any selected video or image.
- **Save Image** – when pressing this button, a frame from the video is captured and added as a separate entity on the left side of the Video Selection panel screen. Notice that to save an image, the video must first be paused.
- **Export** – a button that allows exporting a recorded video. Pressing opens a pop-up menu with a window for entering the export destination by pressing **Add New**.

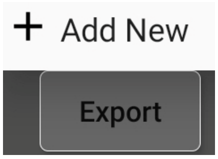

Figure 4-21. Export button

To add an export destination:

a. Press the **+ Add** button on the top right side of the window. The Add folder window appears.

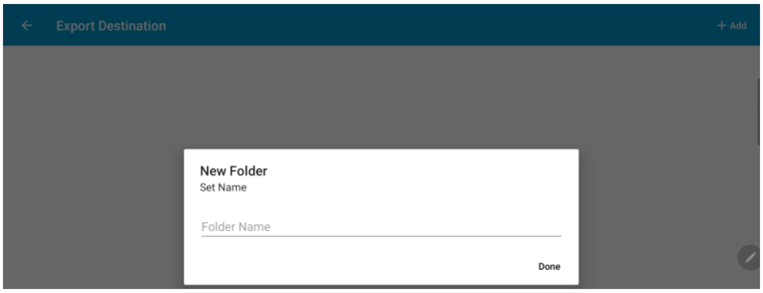

Figure 4-22. Add folder

b. Set the folder **Name** and press **Done**.

c. Click the return ← button at the upper left side of the screen to return to the Review screen.

Next time the Export button is pressed, the added folder name appears on the list, and when selected, the clips are exported to the selected destination folder.

CHAPTER 5

Ultrasound Scanning Operations

Chapter 5 explains the operations performed during a Cardiac ultrasound scan.

5.1 Acoustic Windows

The acoustic window refers to the optimal area on the subject's body where the probe is placed to get the best possible view of the heart. This area is chosen based on where the ultrasound waves can penetrate with the least obstruction from bones or air-filled cavities, as these can block or distort the ultrasound waves.

Achieving a high quality image is highly dependent on the acoustic window. Therefore, it is essential that the user finds the best acoustic window before following the navigation cues.

The UltraSight Static Guidance provides a recommended initial positioning point for placing the Ultrasound Probe for the selected view.

5.2 Probe Initial Position

According to the selected view, the probe's initial position is provided by the UltraSight Static Guidance.

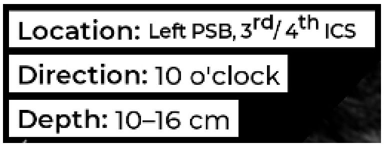

Figure 5-1. Static guide

The above values are the recommendations for the Parasternal Long-Axis view (PLAX).

The recommended initial direction of the probe marker is also indicated by a fixed green arrowhead on the active real-time guidance.

5.3 Probe Direction

The instructions for positioning and manipulating the Ultrasound Probe to the correct location and orientation are according to the probe's direction marking – a dark gray line on the probe.

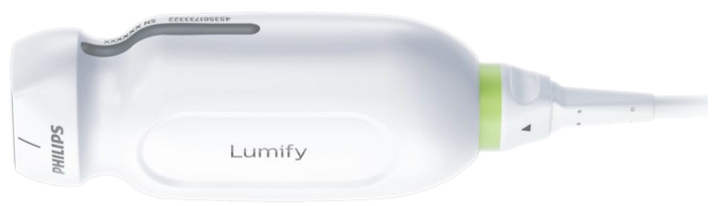

Figure 5-2. Probe direction mark

5.4 Guidance Cues

The UltraSight AI Guidance uses four Dynamic Guidance cues that guide the user on what movements should be performed to reach the correct location and angle of the Ultrasound Probe to achieve an optimal view.

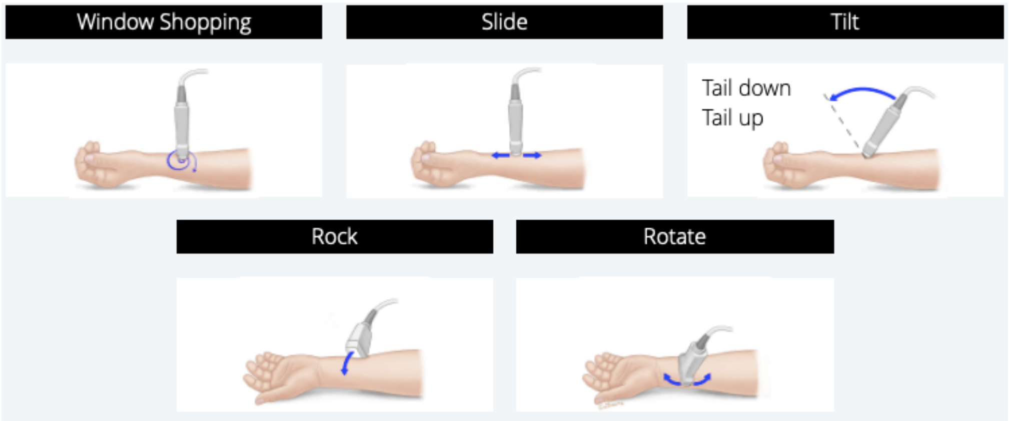

Figure 5-3. Probe manipulations

- **Slide** – slide the probe along one of the four directions, maintaining the probe's orientation.
- **Rotate** – rotate the probe around its axis clockwise and counterclockwise, with the probe's head fixed in place.
- **Tilt** – tilt the probe on its long axis, with its head fixed in place.
- **Rock** – rock the probe on its short axis, with its head fixed in place.

5.4.1 Slide

Slide the probe so that the crosshairs on the image merge with those of the probe.

> *Note*
>
> Slide the probe towards the image crosshairs. The moving part on the screen image is the image crosshairs. The probe crosshairs remain fixed in place.
>
> *Note*
>
> *Slide guidance is not supported for the Subcostal views.*

The moving part on the screen is the image crosshairs. The probe crosshairs remain fixed in place.

The crosshairs show the user the direction to slide the probe.

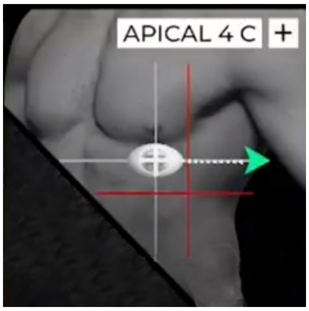

Figure 5-4. Target and probe crosshairs

In the above scenario, slide the probe down and right.

As the probe gets closer to the target, the crosshairs' color change from red to yellow, where the probe's crosshairs and the target's crosshairs merge.

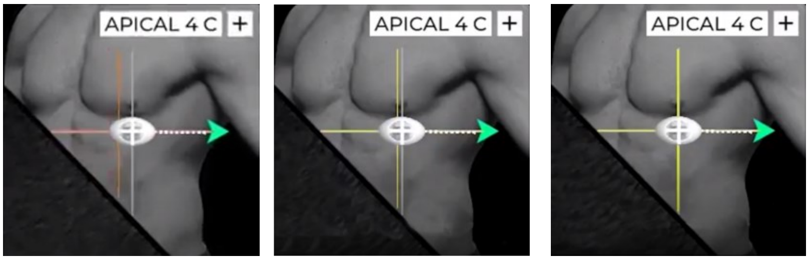

Figure 5-5. Sliding the probe

5.4.2 Rotate

Rotate the probe so that the dotted line, which depicts the probe's axis, rotates towards the green arrowhead.

The moving part on the screen image is always the dotted line, while the green arrowhead remains fixed in place.

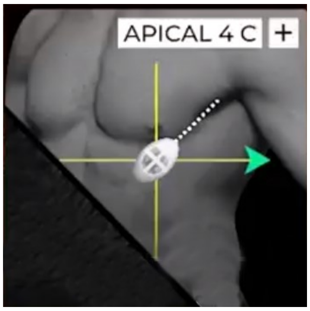

Figure 5-6. Rotate

In the above scenario, rotate the probe in a clockwise direction.

5.4.3 Tilt

Hold the probe against the patient's body while the probe's head remains fixed on the patient's body. Next, tilt the probe tail until the probe is perpendicular to the image crosshairs.

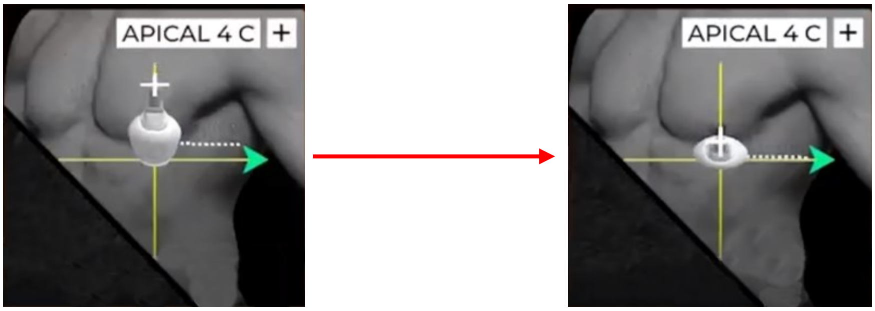

Figure 5-7. Tilt

In the above scenario, tilt the probe's head up.

5.4.4 Rock

Hold the probe against the patient's body while the probe's head remains fixed on the patient's body. Then, rock the probe tail until the probe is perpendicular to the image crosshairs.

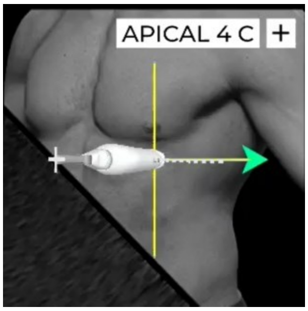

Figure 5-8. Rock

In the above scenario, rock the probe's head to the left.

5.4.5 Probe Guidance Interpretation

Guidance Image	Manipulation Instruction
Sliding	
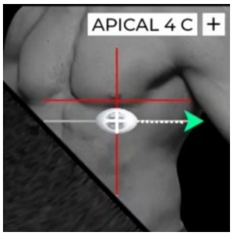	Slide Up
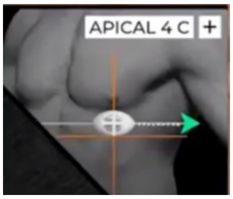	Slide Down
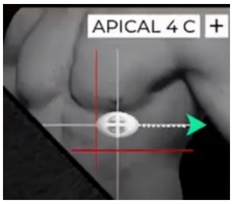	Slide Left and down

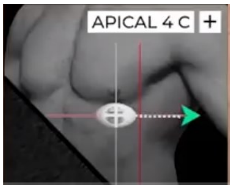	Slide Right
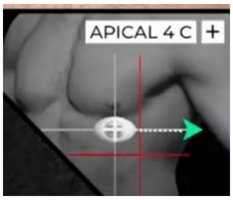	Slide down and right
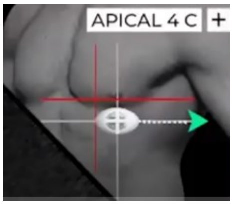	Slide up and left
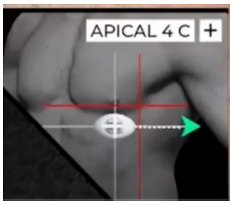	Slide up and right
Rotation	
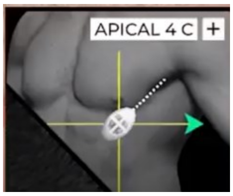	Rotate Clockwise
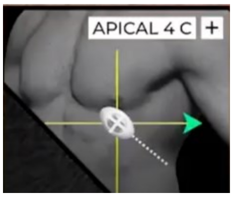	Rotate Counterclockwise
Tilting	
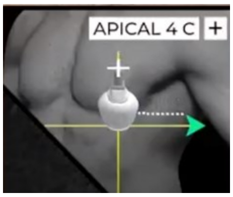	Tilt Up

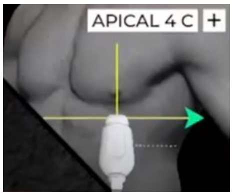	Tilt Down
Rock	
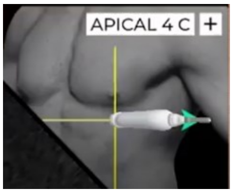	Rock Right
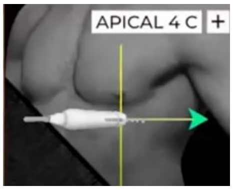	Rock Left

5.5 View Detection

This View Detection provides a real-time indication which differentiate between 3 states.

- No heart:
  The heart is not seen. A text message “Heart not detected” appears.
- Navigate:
  The heart is seen and the quality bar is below 8 bars.
- Hold position:

  When the software calculates that the current view meets diagnostical quality and can be acquired, the following cues are shown:
  - Probe tail is green.
  - Image crosshairs are green.
  - The Quality bar has a minimum of one green bar.
  - “Hold position” message is displayed.

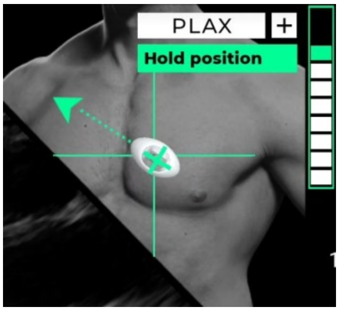

Figure 5-9. Hold position

5.6 Quality Bar

The system provides real-time feedback on the image quality prediction for the current probe position, compared to the ideal position for the selected view of the heart, and advises the user to hold the position to save a clip.

This information is presented on a Quality Bar, indicating how close they are to capturing a diagnostic-quality image.

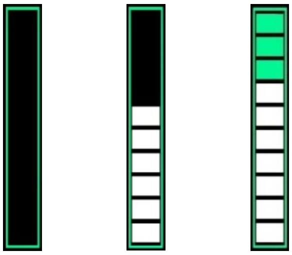

Figure 5-10. Quality bar

The quality bar is a 10-level marker bar that can be filled with up to 10 bars: the bottom 7 bars are white and the upper 3 bars are green. The first green bar represents the threshold for high quality diagnostic image. The number of bars in the Quality bar increases and decreases based on the score for image diagnosability of the current acquired image for the view being scanned.

The user is instructed to record a clip with the highest possible quality bar that they manage to reach for the target view. A higher quality bar indicates that the image is of a higher quality.

5.7 View Optimization

To optimize the view and the UltraSight software performance, the user should adjust the optimal **Gain** and **Depth** settings from within the Lumify user interface, change the patient's position, and request the patient to control their respiration.

> *Reference*
>
> Refer to the Philips Lumify user manual for more details on setting the Gain and Depth.

5.7.1 Depth

The recommended Depth depends on the selected view, patient size, and heart anatomy and appears on the screen.

5.7.2 Gain

The recommended initial Gain for all the views is between 50 and 60. Adjust the Gain to the correct range before starting to scan.

The Gain may need to be changed during the scanning process.

5.7.3 Patient Position

In Apical and Parasternal views, obtaining quality ultrasound video clips of the heart is performed with the patients lying on their left side.

For the Subcostal views, the patient should be lying on their back.

5.7.4 Respiration

Patient respiration movement may affect the quality of the obtained video clip. So once the optimal probe position is obtained, you may request the patient to hold their breath for a couple of seconds while acquiring the video loop.

CHAPTER 6

Scanning with UltraSight Guidance

Chapter 6 guides you through the procedures commonly used when performing patient exams with the UltraSight guidance system.

These procedures include preliminary preparations, entering patient data, using the relevant features of the Phillips Lumify, and acquiring images with the UltraSight AI Guidance software guiding assistance.

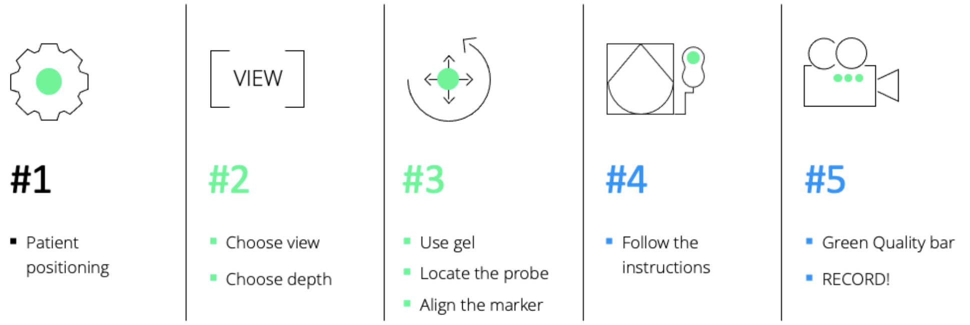

Figure 6-1. Recommended workflow for optimal scan views

> *Reference*
>
> Refer to the Philips Lumify user manual (section 6) for more details on initiating a cardiac scan.

6.1 Preliminary Preparations

1. Verify that the device is charged as needed to complete the exam.
2. Have the ultrasound gel ready for use.
3. Adjust the environment:
    - Set the room temperature to the patient's and user's convenience.
    - Dim the room lights as needed.
4. Position the patient on the exam bed.

6.2 Basic Workflow

This section describes the basic recommended workflow.

1. Launch the UltraSight application.
2. Start a Cardiac exam via the Lumify user interface.
3. Enter the Patient data via the Lumify user interface.
4. Switch the Guidance switch to the On position, or verify it is On.
5. Select the required cardiac View from the View menu.
6. Position the patient in the appropriate position using the Static guidance and the Tutorial window.
7. Set the required Gain and Depth via the Lumify user interface using the Static guidance and the Tutorial window.
8. Apply ultrasound Gel to the probe as needed.
9. Position the Probe in the estimated location and direction for the required view (using the Static Guidance and the Tutorial window).
10. Once the Guidance cues are active (Dynamic Guidance), follow the instructions in all axes: Slide, Rotation, Tilt, and Rock.
11. When the Guidance parameters are positive (Probe tail and image crosshairs are green) and the Quality bar is in its green level (filled with 8 bars or more), the current image quality is at its best and is recommended for saving. In case you cannot reach the Quality Bar green level push the Quality Bar as high as possible, before saving the clip.
12. Save the media via Save Loop on the Lumify user interface (Clip duration is set within the Lumify user interface).
13. Continue acquiring all the required cardiac views.
14. Select End Exam to end and save all the acquired views.

6.3 Starting a New Study

1. Launch the UltraSight application.

   The Ultrasound application opens automatically (if it is not already open) with the UltraSight software overlay.

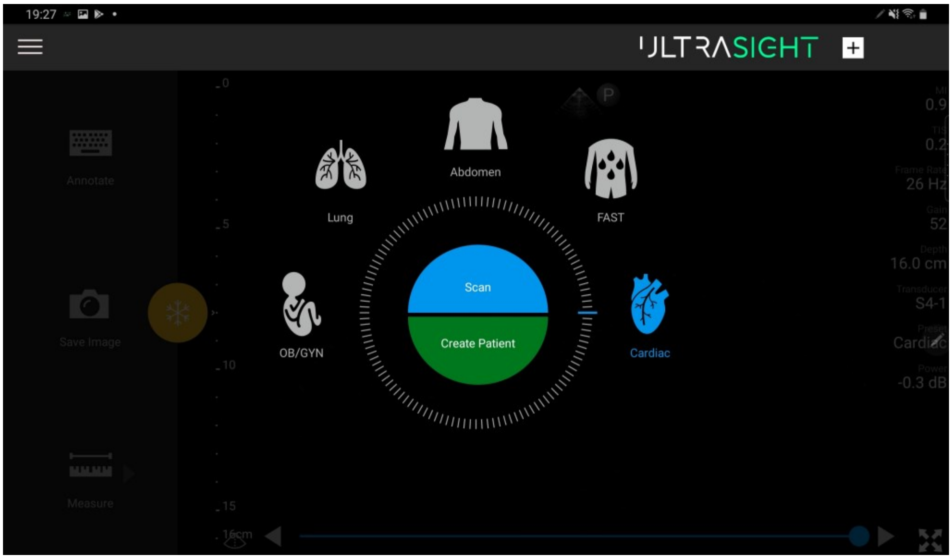

Figure 6-2. Opening screen

> *Note*
>
> If the cardiac probe is not connected to the tablet USB-C port, the Cardiac scan mode is not available.

6.4 Creating a Patient

Creating a patient is performed through the Ultrasound system.

> *Reference*
>
> Refer to the Philips Lumify user manual (chapter 6) for more details on Editing patient data.

> *Caution*
>
> The patient’s information cannot be edited after ending the exam. After ending the exam, you can only view patient information.

6.5 Preparing the Exam

1. Select the **Cardiac** scan type via the Lumify user interface.
2. Switch the **Guidance** button to the **On** position.
3. Select the required cardiac view from the **Views** menu.
4. Set the **Gain** and the recommended **Depth** for the selected view via the Lumify user interface.

6.6 Preparing the Patient

> *Note*
>
> Proper patient position and orientation provide a better sonographic and acoustic window. There is a recommended patient position and orientation for each acoustic window.

1. Position the patient in the appropriate position.
2. Place the device near the patient's head on their left or right.
3. Apply an appropriate amount of ultrasound gel on the U/S probe to achieve a clear and good echo image.

6.7 Performing the Exam

1. Place the tablet on a stable platform at a distance; it can be easily operated in a landscape orientation and facing the examiner.

> *Note*
>
> After placing the tablet, check that you can comfortably reach and touch the tablet screen as needed.

2. Sit comfortably on a chair or the patient's bed.
3. Position the patient in the appropriate position for the selected view.
4. Adjust the **Gain** and **Depth** according to static guidance.
5. Position the U/S probe on the patient's body at the estimated location of the acoustic window and in the recommended direction for the selected view.

Note

See Appendix A for the patient's position for the window views, the depth to set, and the probe positioning. Use the Static Guidance.

Figure 6-3 demonstrates the Ultrasound image at the beginning of an exam and the Static Guidance for the selected view.

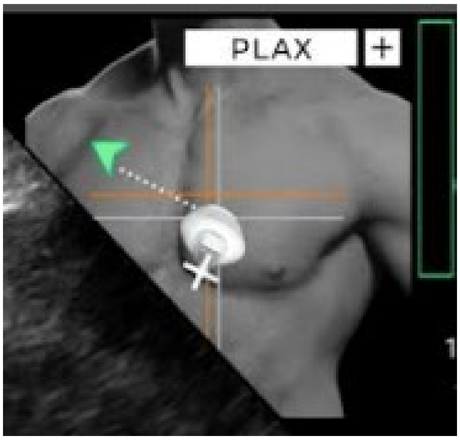

Figure 6-3. Acoustic Window instruction

6. Follow the Static Guidance assistance and the Tutorial window for the Probe **Location** and **Direction** and the recommended **Depth**.
7. Window-Shopping maneuver:

   The window-shopping maneuver is intended to assist the user in locating the appropriate acoustic window for the selected view.

   a. Place the probe at the recommended position and direction on the patient's chest (Para-sternal, Apical, or Sub-costal).
   b. Swipe the probe in a circular motion to find the beating heart.
   c. Once the beating heart is found and the Dynamic Guidance cues are active, follow the UltraSight AI Guidance on the suggested probe manipulations: Slide, Rotation, Tilt, and Rock.

   Figure 6-4 shows the Dynamic Guidance instructing the user to manipulate the probe to the required position and orientation.

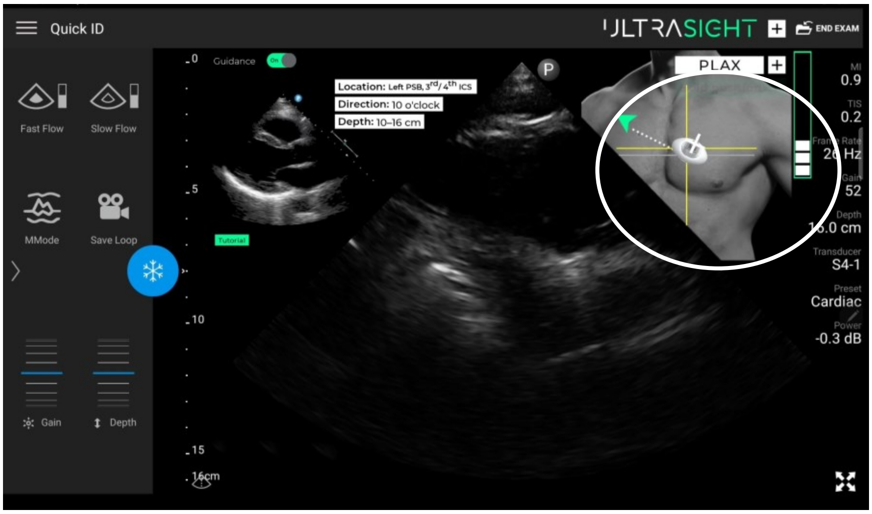

Figure 6-4. Tilt instruction

8. Manipulate the probe marker as recommended by the guiding instructions, searching for the best acoustic window.
9. Adjust the **Gain** and **Depth** again, if needed, to visualize all relevant anatomical structures
10. When the software calculations detect that the current view meets the diagnostical quality of the target view and can be acquired, the probe's tail turns green, the "Hold position" text appears in a green box and the quality bar is loaded with at least one green bar, as shown in Figure 6-5.

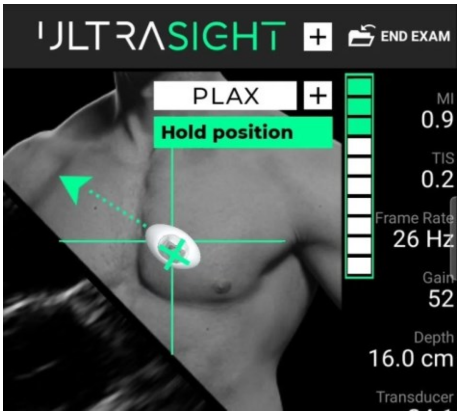

Figure 6-5. Quality bar with a high score

6.8 Acquiring Loops

When the Dynamic Guidance shows that the required position is reached and the Quality Bar is at its peak (8 bars or more), press the Lumify user interface **Save Loop** icon. If eight bars are reached, try to further increase the quality bar if possible, using minor adjustments. If the quality bar doesn't further improve, acquire the image at the current green level. When the green level is not reached after following the guidance instructions, push the quality bar as high as possible before saving the clip.

Wait until the icon turns yellow and a Loop Saved notification is displayed.

> *Reference*
>
> For more information about acquiring loops and setting the loop duration, see Chapters 3 and 6 in the Philips Lumify user manual.

6.9 Continuing and Completing the Exam

1. Continue acquiring all the required cardiac views.
2. Select **End Exam** to end and save all the acquired views.

6.10 Reviewing Acquired Loops

1. Press the Lumify Quick ID menu button.
2. Press **Review Exams**.

> *Reference*
>
> See Chapter 7 in the Philips Lumify user manual for more information about reviewing an exam.

CHAPTER 7
Clinical Studies

7.1 Pivotal Study

A multi-center prospective study was conducted to assess the quality of echocardiographic clips obtained by medical professionals without specialized echocardiography training using the UltraSight AI Guidance software.

7.1.1 Pre-Study Phase

Six registered nurses (RN) and three medical residents underwent an 8-hour training session, which included theoretical and hands-on training developed by the sponsor for performing echo exams using UltraSight AI Guidance.

7.1.2 Study Phase

Adult males and females underwent two 2D Limited-TTE exams with the Lumify Ultrasound system: One by the RN/medical resident user using UltraSight AI Guidance and a second exam by a sonographer without the AI Guidance.

In each echocardiography exam, 10 standard views were captured by the RN/medical resident and the sonographer: PLAX, PSAX-AV, PSAX-MV, PSAX-PM, AP4, AP5, AP2, AP3, SC-4C, and SC-IVC. Each RN/medical resident user scanned 23–30 subjects.

7.2 Exams' Quality Evaluation By Expert Cardiologists

The patients' exams acquired by the RN/medical resident users and the echocardiography sonographer were given to five expert cardiologists, who were blinded to the operator that acquired the exams, to the assessments of other panel members, and to the site where the exams were obtained.

The expert cardiologists assessed whether each exam, taken as a whole, was of sufficient quality (Yes/No) to evaluate each of the four predefined primary endpoints.

The expert cardiologists specifically evaluated whether the exams permitted qualitative visual assessment of left ventricular (LV) size, LV function, right ventricular (RV) size, and non-trivial pericardial effusion. They also evaluated the diagnostic quality of six additional anatomical elements and the quality of the ten echocardiographic views.

The expert cardiologists' evaluations of the exam quality followed the American College of Emergency Physicians (ACEP) "Suggested Quality Assurance Grading Scale" for echocardiography quality. According to this scale, the minimum score to define a clip as diagnostic is Grade 3 or above.

7.3 Primary Endpoint

Based on a majority (at least 3) of the 5 expert cardiologists, the exams performed by the RNs/medical residents had sufficient visual quality in 93–100% of cases to assess LV size and function, RV size, and pericardial effusion.

Subject BMI, age, gender, and cardiac abnormality status had no effect on the sufficient quality to assess LV size and function, RV size, and pericardial effusion when RNs/medical residents performed the exam.

The number of exams (1–14 vs. 15 or more) performed by the RNs/medical residents and their profession also had no effect on sufficient quality to assess LV size and function, RV size, and pericardial effusion. Additionally, the clinical site did not affect the quality of these assessments. The null hypothesis for the four primary endpoints was that the percent of diagnostic quality is 80% or less versus the alternative that it is above 80%. The lower bound of all CIs is above 80%, and hence all 4 endpoints were successful.

Table 1. Primary endpoints results

	Percent of diagnostic quality (95% CI)
Qualitative Visual Assessment of LV size	99% (99–100%)
Qualitative Visual Assessment of LV function	100% (97–100%)
Qualitative Visual Assessment of RV size	93% (89–99%)

	Percent of diagnostic quality (95% CI)
Qualitative Visual Assessment of Pericardial effusion	100% (98–100%)

7.4 Secondary Endpoints

Based on a majority (at least 3) of the 5 expert cardiologists, the exams performed by RNs/medical residents had a sufficient visual quality to assess MV structure, RV function, left atrium size, and AV structure in 89–98% of cases and to assess TV structure and IVC size in 74% and 67% of cases, respectively. Furthermore, the exams performed by RNs/medical residents had sufficient diagnostic quality to assess the 10 views: PLAX, PSAX-MV, PSAX-PM, AP2, AP3, AP5, and SC-4C in 81–94% of cases, and to assess PSAX-AV and SC-IVC in 72% and 68% of cases, respectively.

Table 2. Secondary endpoints results

	Percent of diagnostic quality (95% CI)
Qualitative Visual Assessment of RV Function	94% (90–98%)
Qualitative Visual Assessment of Left atrium size	94% (90–97%)
Qualitative Visual Assessment of AV structure	89% (84–93%)
Qualitative Visual Assessment of MV structure	98% (95–99%)
Qualitative Visual Assessment of TV Structure	74% (67–80%)
Qualitative Visual Assessment of IVC size	67% (59–75%)

AV = aortic valve, IVC = inferior vena cave, LV = left ventricular, MV = mitral valve, RV = right ventricular, TV = tricuspid valve

Table 3. Secondary endpoints results (of the 10 views)

	Percent of diagnostic quality (95% CI)
PLAX	86% (79–93%)
PSAX-AV	72% (65–79%)
PSAX-MV	88% (83–92%)
PSAX-PM	90% (85–96%)
AP4	94% (90–97%)

	Percent of diagnostic quality (95% CI)
AP2	85% (78–90%)
AP3	83% (76–91%)
AP5	87% (80–95%)
SC- 4C	81% (73–89%)
SC- IVC	68% (61–75%)

AP2 = apical 2-chamber, AP3 = apical 3-chamber, AP4 = apical 4-chamber, AP5 = apical 5-chamber, AV = aortic valve, LV = left ventricular, PLAX = parasternal long axis, PSAX-AV = parasternal short axis aortic valve, PSAX-MV = parasternal short axis mitral valve, PSAX-PM = parasternal short axis at the papillary muscle, SC-4C = subcostal 4-chamber, SC-IVC = subcostal inferior vena cava

Note: Secondary endpoints were not evaluated based on a specific hypothesis. Since the evaluation of secondary endpoints did not allow for control of Type I error, the related study results are presented as a descriptive demonstration of the use of UltraSight AI Guidance for the specific secondary endpoints.

7.5 Safety Profile

No adverse events were reported in the pivotal study, which demonstrated the safety of UltraSight AI Guidance software.

7.6 Human Factors Validation Study

The UltraSight AI Guidance user interface and training materials were tested during the Human Factors Validation Study. The study was conducted with a total of 34 participants to demonstrate the usability of the device.

The study enrolled five (5) user groups of healthcare professionals:

- Physicians (Hospitalists): 6 users
- Registered Nurses (RNs): 17 users
- Medical Residents: 5 users
- Certified Medical Assistants: 6 users

The study demonstrated that 100% of critical tasks passed across all user types. No use errors were found that could cause harm for the patient or the scanner. Given the same level of training, healthcare professionals across the backgrounds that were tested performed consistently during independent scans with UltraSight AI Guidance on a patient model.

This study has demonstrated that the UltraSight guidance software can be used safely by the intended users, for the intended uses, in the intended use environments.

7.7 Non-Clinical Testing

The following performance testing was conducted to evaluate the device performance:

1. Software moderate level of concern verification and validation testing was conducted as required by IEC 62304 and FDA Guidance on General Principles of Software Validation, January 11, 2002.

2. UltraSight AI Guidance Algorithms' standalone performance was validated with a number of standalone performance tests, see detailed description below.

3. Human factors Validation as detailed in Section 7.6 above.

Non-Clinical Standalone Performance Testing of AI algorithms

The Datasets used for algorithm development and performance testing are listed in Table 4:

Table 4: Data used for Algorithm Development and Performance Testing

**Phase**	**Algorithm development**	**Performance tests for Quality Bar**	**Performance test for View Detection and Guidance tests**
**Number of subjects**	580	312	75
**Number of samples**	5 million frames of ultrasound images	5,800 clips	2.3 million frames of ultrasound images

The data used for performance testing was collected at different sites, geographically separated, from the sites used for collection of the algorithm development data. The data used in the performance testing was collected from a population representative to the intended population.

Performance Tests Overview

The following features of the system were tested:

- Quality bar

- View detection (part of the quality bar functionality as explained below)
- Probe Guidance

Each feature of the system (Quality Bar, View Detection, Probe Guidance) was tested independently.

Quality Bar:

The quality bar is a score for image diagnosability. It represents the classification between high- and low-quality images, where high quality images are defined as grade 3 or more based on ACEP guidelines.
The test was performed on 312 clips. Each clip in the dataset is associated with a target cardiac view and was annotated with a clip diagnosability label (diagnosable / non-diagnosable), by three cardiologists.

The test assessed the classification performance between “diagnosable” and “non diagnosable” clips of each view. The mean AUC was 0.86 with 95% CI [0.85, 0.87] showing good classification performance, relative to the success criteria of AUC > 0.8. The mean PPV was 0.93 with 95% CI [0.92, 0.94] relative to the success criteria of PPV > 0.75, showing good classification performance. Stratified analysis was performed, for each target cardiac view the AUC was greater than 0.8, and the PPV was above 0.75.

View Detection:

The system provides view detection classification that distinguishes between three system states: “Hold Position", “Navigate", and “No Heart". The test was performed on 75 subjects on a total of 2.3 million frames of ultrasound images.

The view detection classification performance is quantified for the classification tasks of “Hold position" vs. “Navigate" and “Hold position" vs. “No heart". The ground truth labels were defined on the frame level using annotation of expert sonographers. The mean AUC was 0.988 with 95% CI [0.985, 0.990] showing good classification performance, relative to the

success criteria of AUC > 0.8. Stratified analysis was performed and showed that for each individual binary classification test the AUC was above 0.8.

Probe Guidance:

The system provides guidance cues when the heart is visible on the ultrasound image. To provide the correct guidance cues, the system needs to be able to identify the position and orientation of the probe relative to the target cardiac view. There are five possible guidance cues: rotations, tilts, rocks, and slides in the x (lateral-medial) and y (superior-inferior) directions.

The testing was performed on 75 subjects on a total of 2.3 million frames of ultrasound images. The testing evaluated the frame level accuracy of each guidance cue prediction. For each guidance cue, a series of tests was defined to check if the guidance cue is functioning correctly in a particular direction (for example, clockwise rotations relative to 2C view, tilts up from PLAX). Each test was formulated as a binary classification test.

The mean AUC was 0.821 with 95% CI [0.813, 0.827] showing good classification performance, relative to the success criteria of AUC > 0.8. Stratified tests show that individual classifiers have reasonable discrimination ability, and acceptable classifiers.

CHAPTER 8
Troubleshooting

8.1 Introduction

The following section addresses only the UltraSight application troubleshooting.

> *Reference*
>
> See Chapter 9 in the Philips Lumify user manual for Lumify system troubleshooting.

8.2 UltraSight Application Troubleshooting Guide

Following are possible scenarios that may occur while performing an exam and how to solve the issue.

8.2.1 Heart Not Detected

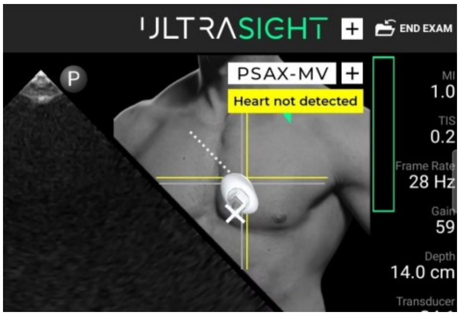

Figure 8-1. Heart not detected

If the "**Heart not detected**" message appears, do the following:

1. Check that the displayed view is the required view.
2. Verify the appropriate patient position.
3. Verify that the probe location and direction are as recommended by the static instructions.
4. Check that the Depth is set to the recommended value.
5. Check that the Gain is appropriate.

6. Verify that there is sufficient gel on the U/S probe's head.
7. Move the probe on the patient's chest (Window shopping maneuver) to find the optimal acoustic window.
8. Ask the patient to hold their respiration to assist with detecting the beating heart.

8.2.2 Verify Position Depth and Gain

If the "**Verify position depth and gain**" message appears, gentle adjustments are required to acquire a satisfying image.

1. Make sure the probe is stable on the patient.
2. Ask the patient to hold their respiration as needed to assist with detecting the beating heart.
3. Check that the probe is positioned correctly and anchored to the patient.
4. Ask the patient to take a deep breath and hold it.
5. Check that the Depth is set to the recommended d value.
6. Check that the Gain is appropriate.
7. Verify that there is sufficient gel on the U/S probe's head.

8.2.3 Low-Quality Indication without Guidance Cues

If the quality bar is empty and without guidance cues, follow and review the protocol for acquiring an image:

1. Acquire a relevant basic view of the current acoustic window.
2. Once acquired, select the requested view, and follow the navigation tool instructions.
3. Verify that the Depth is suitable.
4. Guide the patient with their Respiration.
5. Manipulate the Probe's angle – point the probe towards the location of the heart.
6. Improve the Probe's position by rolling the patient on their side.

CHAPTER 9
Appendices

Appendix A. Windows and Views

A.1. Parasternal Window

The PARASTERNAL window includes 4 different views. Scanning at these views is performed with the probe at almost the same location.

The recommended Depth for the PARASTERNAL view is 12–16 cm.

The view depends on the patient's anatomy and should be adjusted per patient so that the entire heart image is visible. Start in the middle of the range and adjust as necessary.

The patient position for the Parasternal views is left decubitus – Position the patient per the recommended view, with the patient laying on their left-hand side with the lower arm tucked under the head and knees bent for comfort.

A.1.1. PLAX View

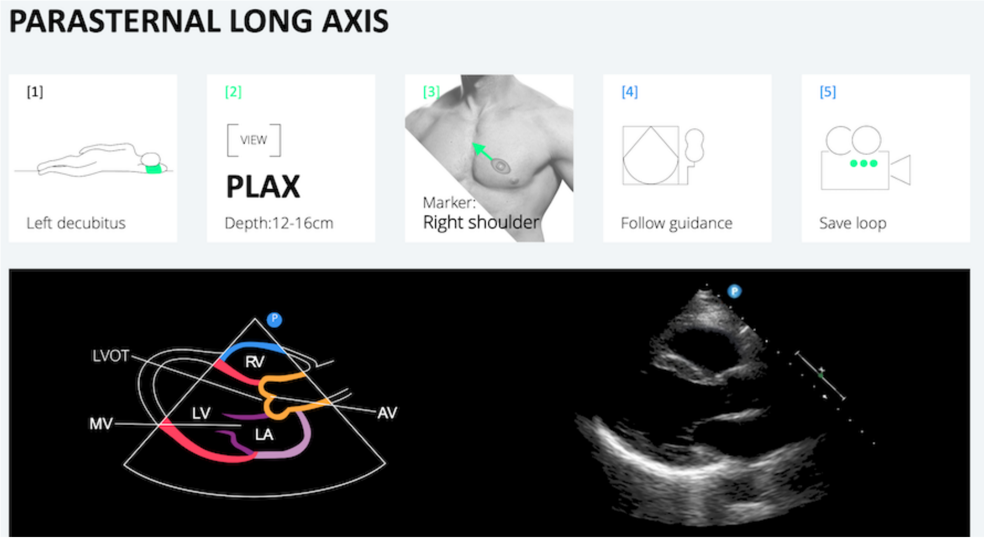

Figure 9-1. PLAX view

A.1.2. PSAX-AV View

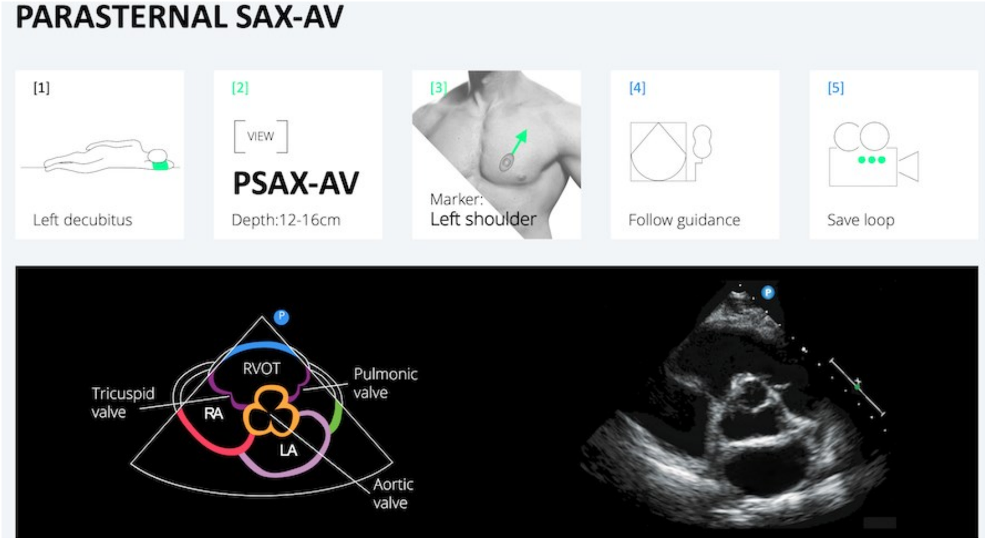

Figure 9-2. PSAX-AV view

A.1.3. PSAX-MV View

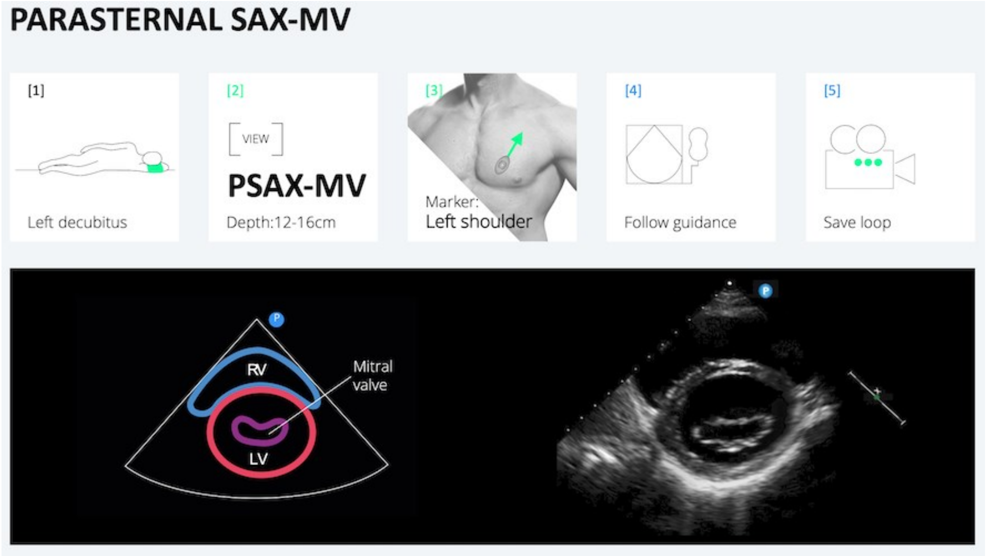

Figure 9-3. PSAX-MV view

A.1.4. PSAX-PM View

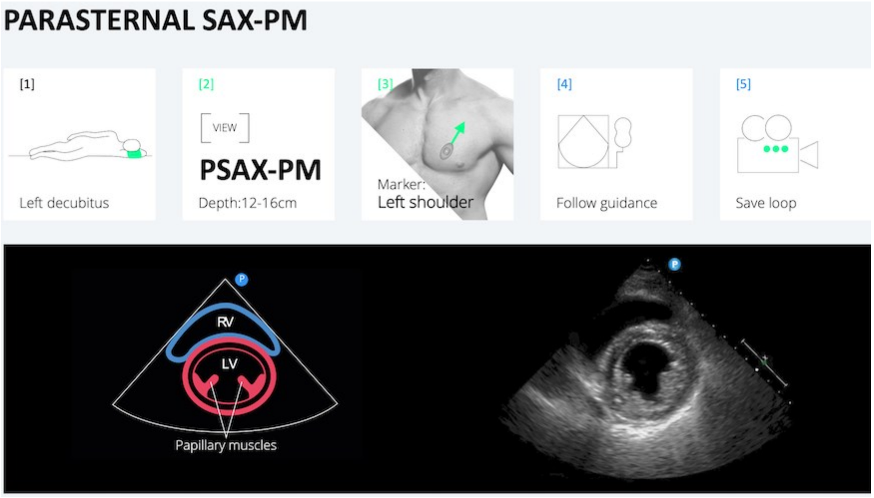

Figure 9-4. PSAX-PM view

A.2. APICAL Window

The APICAL window includes 4 different views. Scanning at these views is performed with the probe at almost the same location.

The recommended Depth for the APICAL window is 14–18 cm.

The view depends on the patient's anatomy and should be adjusted per patient so the entire heart image is visible. The view also depends on the patient's breathing; a deep inhalation or deep exhale may be required to acquire the best image possible.

The patient position for the Apical views is left decubitus – Position the patient per recommended view, with the patient laying on their left-hand side with the lower arm tucked under the head and knees bent for comfort.

A.2.1. APICAL 4C View

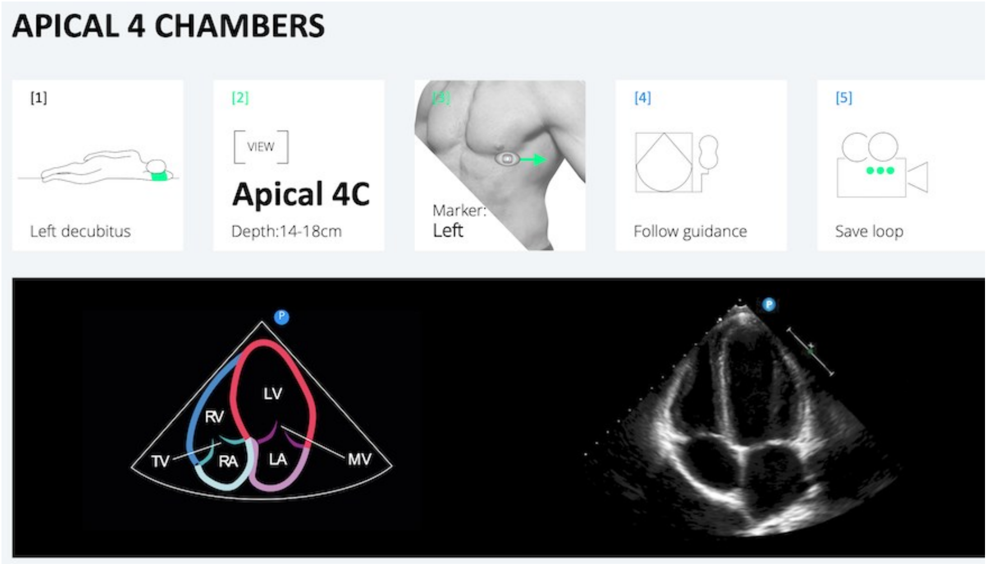

Figure 9-5. APICAL 4C view

A.2.2. APICAL 3C View

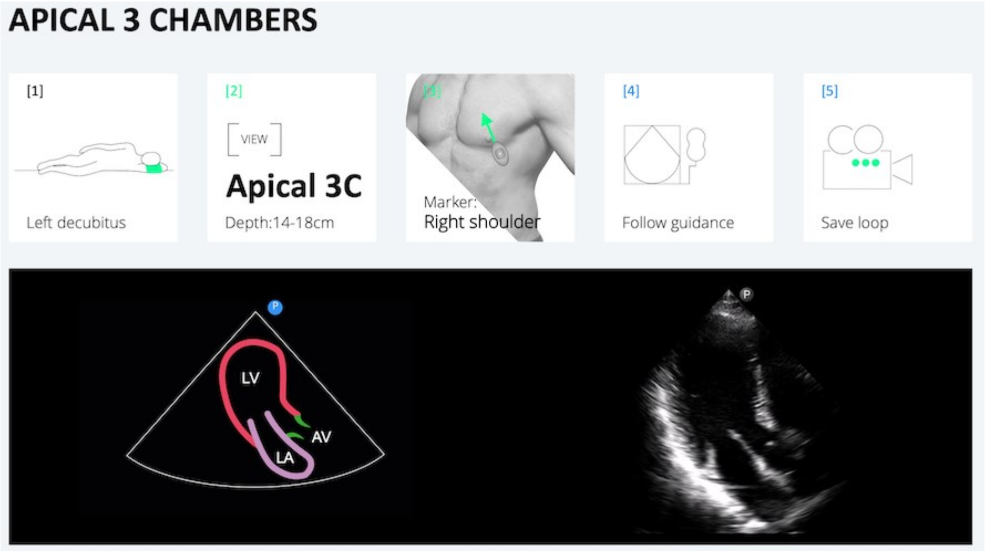

Figure 9-6. APICAL 3C view

A.2.3. APICAL 2C View

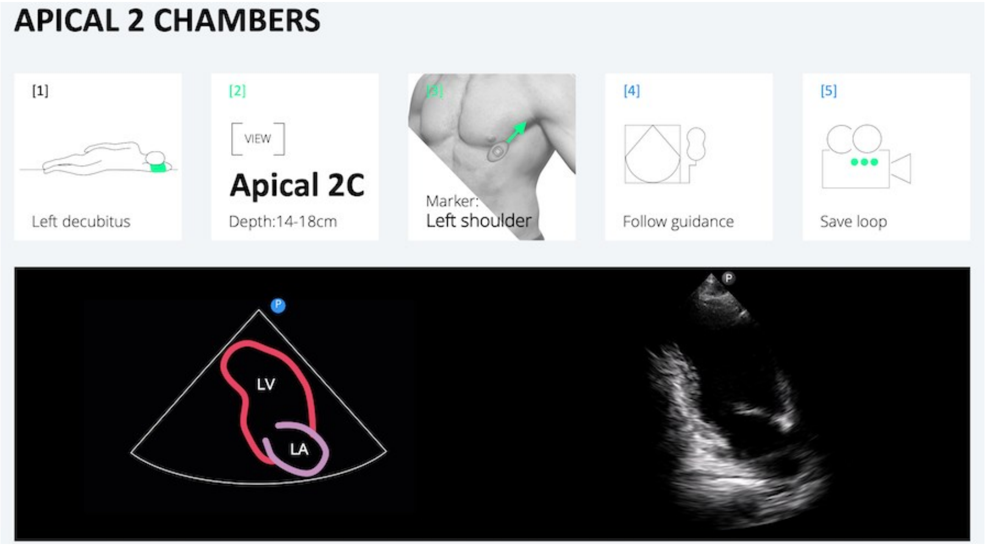

Figure 9-7. APICAL 2C view

A.2.4. APICAL 5C View

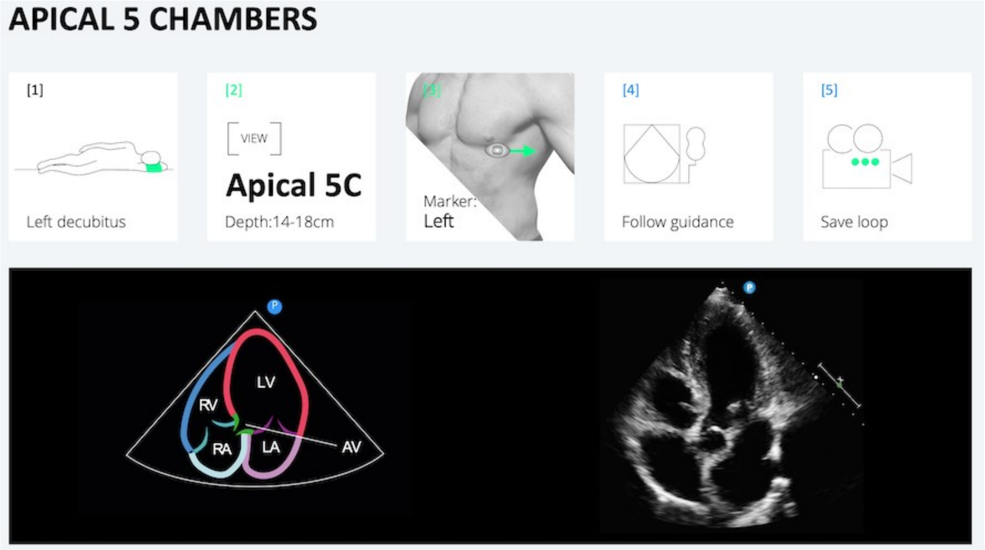

Figure 9-8. APICAL 5C view

A.3. SUBCOSTAL Window

The SUBCOSTAL window includes 2 different views. Scanning at these views is performed with the probe at almost the same location.

The SUBCOSTAL view is deeper than the previous two views and requires a change in the patient's position.

The recommended Depth for the SUBCOSTAL set is 18–24 cm deep. The view depends on the patient's anatomy and should be adjusted per patient so the entire heart image is visible. Adjust the Depth to the middle value. The view also depends on the patient's breathing; a deep inhalation or deep exhale may be required to acquire the best image possible.

Position the patient in a supine position and place the probe at the xiphoid part of the sternum right below the solar plexus. This view requires pushing the probe a bit deeper into the patient's chest.

A.3.1. SUBCOSTAL SC-4C

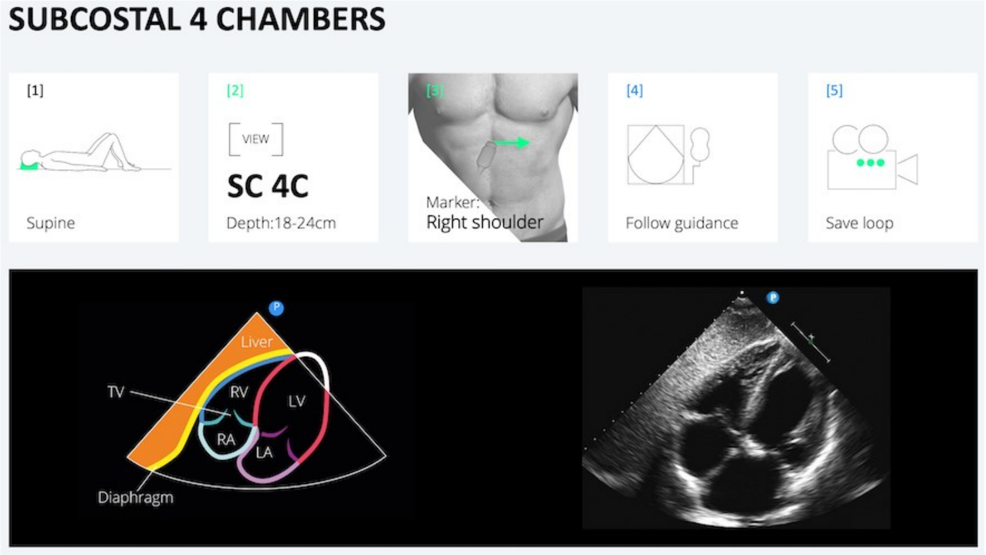

Figure 9-9. SUBCOSTAL SC-4C

A.3.2. SUBCOSTAL SC-IVC

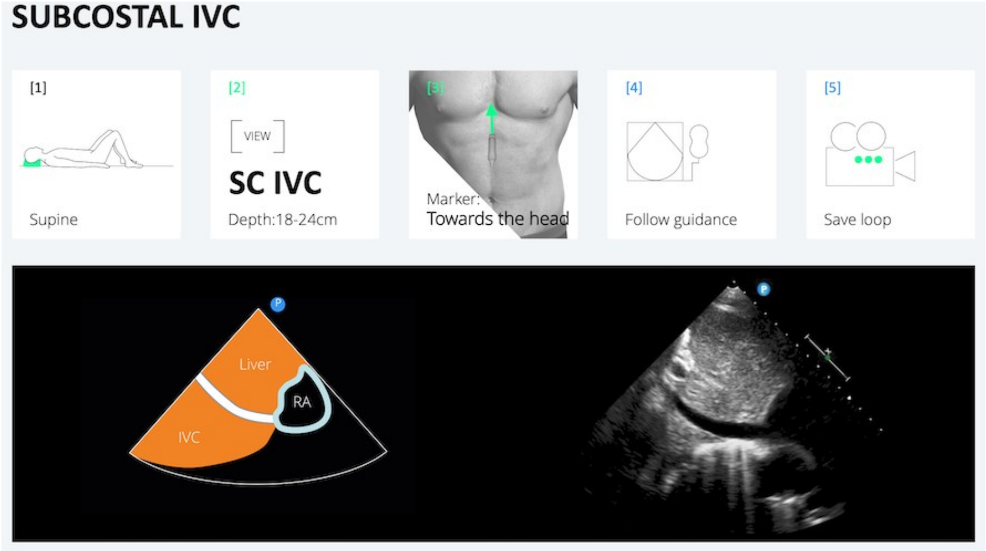

Figure 9-10. SUBCOSTAL IVC-4C

Appendix B. Supported Ultra-Sound Systems

B.1. Philips Lumify Ultrasound System

- Model Name: Philips Lumify; Transducer: S4-1
- Model Number: Lumify Power Module (LPM) Philips, Philips part number: 453561998451
- Philips Lumify, software number/version:
  4.0 453562133201 21-11-16 or 4.0.1 453562163981 22-09-15.

UltraSight Ltd.
UM-0001

June 2024

Email: Info@ultrasight.com
Info: https://www.ultrasight.com/
