# Supplementary material for: Limited echocardiogram acquisition by novice clinicians aided with deep learning: A randomized controlled trial
Source: Biol Methods Protoc. 2025 Nov 7;10(1):bpaf083. doi: 10.1093/biomethods/bpaf083 (PMC12627401; doi:10.1093/biomethods/bpaf083)
Supplement: bpaf083_Supplementary_Data [file bpaf083_supplementary_data.zip › Supplementary_Material.docx]

**Supplementary Figure 1: Detailed Description of Ultrasight System**

The main features of the UltraSight AI Guidance system (Quality Bar, View Detection, Probe Guidance) are implemented using a neural network. The user chooses a target view of the heart, from a list of the 10 supported standard cardiac views.

A user scans a subject using Philips Lumify US probe,. Based on the ultrasound images, the UltraSight AI Guidance guides the user on where to place the transducer and how to manipulate it to acquire an optimal view.

The guidance graphical instructions are continuously updated while the user moves the transducer. The system displays an image quality bar that is continuously updated while the user scans the patient, the user attempts to find the maximal quality.

When the user decides to acquire a clip, they use the Lumify application clip saving functionality to save a clip as in a standard echocardiography exam.

**Panel A** demonstrates that the user is off axis, and the system provides a suggestive corrective maneuver to obtain the optimal image. **Panel B** demonstrates that an optimal view has been obtained.

**Supplementary Figure 2. Modified Rapid Assessment for Competency in Echocardiography (RACE) Tool.**


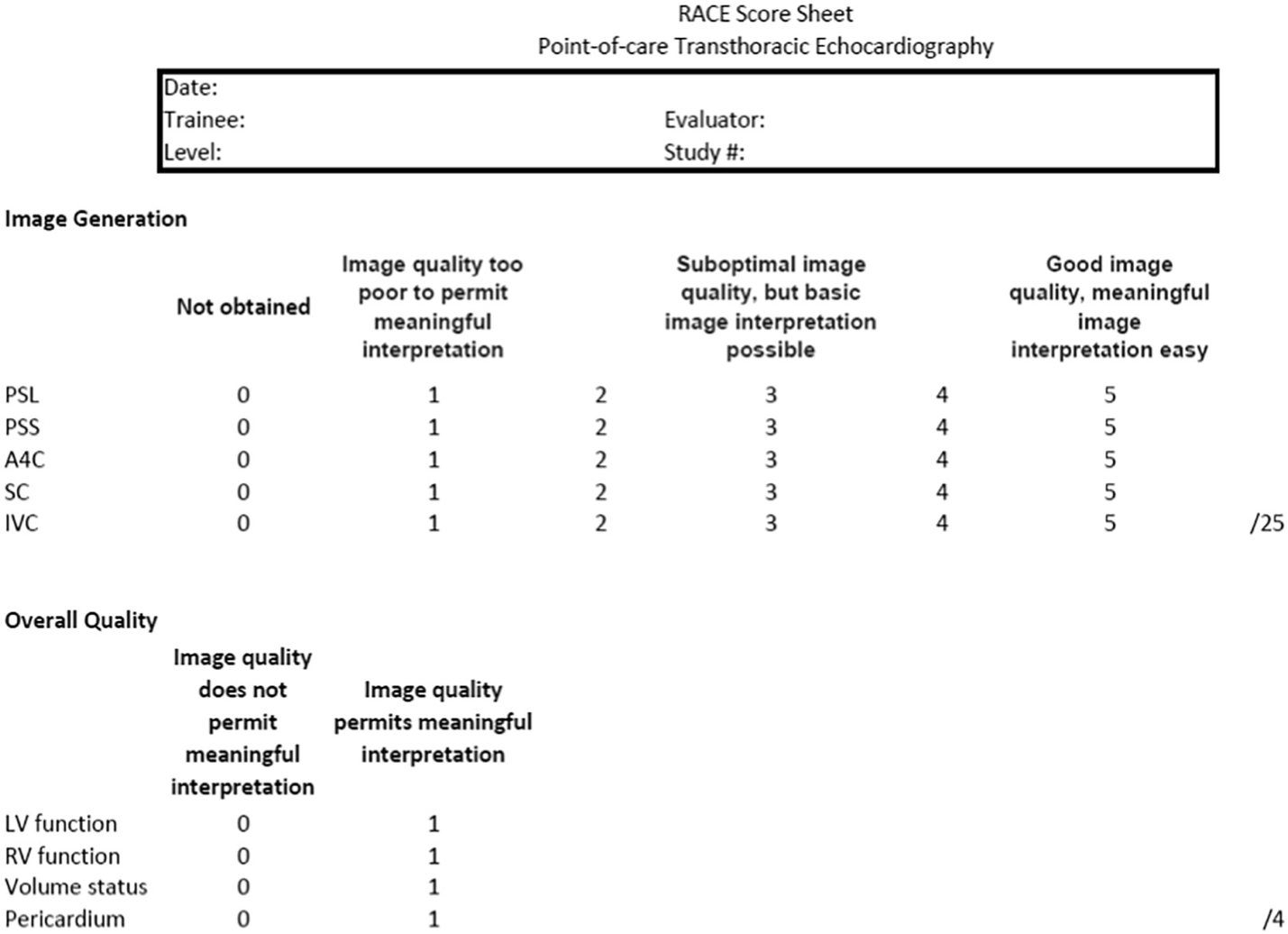


**Supplement 3. Participant Instructions for Assessment and Education.**

This supplement provides a step-by-step description of the participant workflow used in the randomized trial, from orientation to follow-up assessment. All instructional procedures were standardized and delivered by the same study investigator to ensure consistency across participants and study arms.

**Stage I – Orientation to Testing Device**

**Objective:** Familiarize participants with the ultrasound testing device and confirm understanding of basic operational functions.

**Device:** Butterfly iQ+ (used for baseline and follow-up assessments).

**Procedure:**

1. Orient learner to the device interface and controls (“knobology”), including depth, gain, mode selection, and how to save and label views.
2. Do **not** provide instruction on how to obtain specific cardiac views.
3. Assess understanding by **teach-back**, ensuring learners can independently demonstrate adjustment of depth/gain and image saving.
4. No image quality feedback is provided during this phase.

**Stage II – Baseline Assessment**

**Objective:** Evaluate participant baseline ability to obtain five standard echocardiographic views.

**Procedure:**

1. The testing device (Butterfly iQ+) is pre-set to **cardiac mode** and ready for use.
2. The learner is instructed to obtain and save the following five cardiac views:
   - Parasternal Long Axis (PLAX)
   - Parasternal Short Axis (PSAX)
   - Apical Four Chamber (A4C)
   - Subcostal (SC)
   - Inferior Vena Cava (IVC)
3. Learners are told:
   - They may **take as long as needed** to acquire each view.
   - They may **reposition the patient** as necessary.
   - They should **verbally indicate** to the proctor when they begin saving each clip (to time the acquisition).
   - If unable to obtain an adequate image, they may **save their best attempt** or **indicate they are giving up** and proceed to the next view.
4. Assess understanding of instructions by **teach-back** before beginning.
5. Upon completion, the participant is sent an electronic survey link (attitudes and confidence assessment).

**Stage III – Post-Randomization Orientation**

**Objective:** Orient participants to the assigned study device prior to the two-week clinical integration period.

**Device:** Philips Lumify™ (standard for both arms).

**Procedure:**

1. Provide orientation to:
   - Device login and startup.
   - Selecting “Cardiac” mode.
   - Adjusting depth and gain.
   - Saving and labeling images.
2. Assess understanding via **teach-back**.
3. Allow the learner to **practice logging in and performing test scans** on the standardized patient.
4. **No feedback** is given on scanning technique or image quality.

**Stage IIIb – Ultrasight™ Orientation (Intervention Arm Only)**

**Objective:** Introduce participants randomized to the DL-guided arm to Ultrasight software functionality.

**Procedure:**

1. After completing Stage III, introduce the **Ultrasight™ system**.
2. Orient the learner to:
   - Login procedures.
   - Activation of Ultrasight software on the Lumify device.
   - Operation of the real-time guidance overlay.
3. Demonstrate how the system provides directional cues by showing an intentionally **off-axis view** on the standardized patient.
4. Verbally instruct the learner to follow the on-screen guidance to optimize the image.
5. Permit the learner to **practice scanning** using Ultrasight guidance, without performance feedback.
6. Confirm comprehension via **teach-back**.

**Stage IV – Educational Material Distribution**

**Objective:** Reinforce independent learning resources for both study arms.

**Procedure:**

1. Email participants a follow-up message containing:
   - A link to **Stanford 25 POCUS educational materials** (overview of cardiac POCUS acquisition). https://www.youtube.com/watch?v=fsRrC53sWus
   - A link to the **Ultrasight guidance overview** (https://ultrasight.com/about/)for the intervention arm.
